# Materials, Structure, and Interface of Stretchable Interconnects for Wearable Bioelectronics

**DOI:** 10.1002/adma.202408456

**Published:** 2024-08-13

**Authors:** Yue Li, Asmita Veronica, Jiahao Ma, Hnin Yin Yin Nyein

**Affiliations:** ^1^ Department of Chemical and Biological Engineering The Hong Kong University of Science and Technology Hong Kong SAR 00000 China

**Keywords:** flexible materials, metals, soft–rigid interfaces, stretchable interconnects, wearable bioelectronics

## Abstract

Since wearable technologies for telemedicine have emerged to tackle global health concerns, the demand for well‐attested wearable healthcare devices with high user comfort also arises. Skin‐wearables for health monitoring require mechanical flexibility and stretchability for not only high compatibility with the skin's dynamic nature but also a robust collection of fine health signals from within. Stretchable electrical interconnects, which determine the device's overall integrity, are one of the fundamental units being understated in wearable bioelectronics. In this review, a broad class of materials and engineering methodologies recently researched and developed are presented, and their respective attributes, limitations, and opportunities in designing stretchable interconnects for wearable bioelectronics are offered. Specifically, the electrical and mechanical characteristics of various materials (metals, polymers, carbons, and their composites) are highlighted, along with their compatibility with diverse geometric configurations. Detailed insights into fabrication techniques that are compatible with soft substrates are also provided. Importantly, successful examples of establishing reliable interfacial connections between soft and rigid elements using novel interconnects are reviewed. Lastly, some perspectives and prospects of remaining research challenges and potential pathways for practical utilization of interconnects in wearables are laid out.

## Graphical abstract

Stretchable interconnects are one of the fundamental components in achieving robust wearable devices, but their importance is often overlooked. This review highlights their importance and provides insightful information on various materials, configurations, and manufacturing approaches being studied for interconnects. Their practical potentials for wearable bioelectronics are also discussed for future research opportunities.

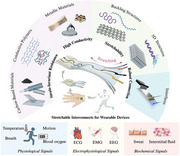

## 1 Introduction

**Figure 1:**
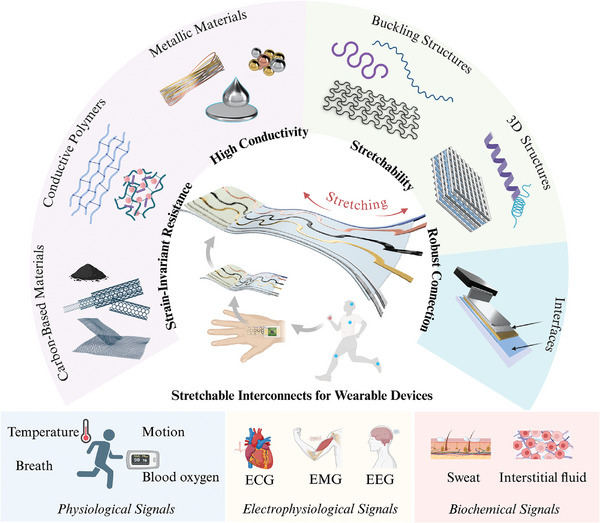
A schematic illustration of the strategies for designing stretchable interconnects in wearable bioelectronic applications.

The rise of wearable technology in the past decade has revolutionized today's healthcare by enabling remote and continuous health monitoring.^[^ ^1^, ^2^ ^]^ Wearable healthcare devices integrate biological and electronic systems in a wearable format to measure and convey important health parameters by capturing subtle biological signals. They encompass physiological (e.g., body temperature,^[^ ^3^, ^4^ ^]^ motion,^[^ ^5^, ^6^ ^]^ breath,^[^ ^7^ ^]^ and blood oxygen,^[^ ^8^, ^9^ ^]^) electrophysiological (e.g., electrocardiograms (ECG),^[^ ^10^, ^11^ ^]^ electromyograms (EMG),^[^ ^12^, ^13^ ^]^ and electroencephalograms (EEG),^[^ ^14^ ^]^) and biochemical (e.g., biomarkers in body fluids^[^ ^15^, ^16^, ^17^, ^18^ ^]^) information to facilitate health wellness to chronic disease management (**Figure** **1**).^[^ ^19^ ^]^ To realize this goal, the reliability of the wearable signals is necessary, and one of its prerequisites is to maintain a conformal and seamless contact between a wearable device and human skin. Given the soft and dynamic nature of the skin, skin analogs possessing elasticity (i.e., stretchability and bendability) along with breathability and biocompatibility are needed for device/skin interface.^[^ ^20^, ^21^ ^]^ Human skins are highly anisotropic. Young's modulus can range from 5 to 140 kPa with a regular skin deformation of 20%, and the deformation may reach over 30% at the joints.^[^ ^1^, ^22^ ^]^ Such dynamic requirements bring challenges to designing a suitable material and structure integrated into wearable devices.

Wearable bioelectronics contain multiple functional components such as sensors, a power source, energy harvesters, flex circuits, and microcontrollers integrated into a single system. To ensure seamless integration on the skin, these components are made to achieve suitable mechanical properties via materials engineering and structural design. Electrical interconnects, which are the fundamental units that connect various functional components in the device, have been underexplored. Although interconnects do not directly involve in signal detection and conversion, their electrical and physical characteristics play an important role in determining the signal's sensitivity, stability, fidelity, and energy efficiency of the entire device. The interconnects require a conductivity that is commonly larger than 1000 S cm^−1^ to minimize signal interference and resistive energy losses.^[^ ^23^ ^]^ In stretchable devices, interconnects should withstand a strain significantly larger (>100%) than the functional counterparts.^[^ ^24^, ^25^ ^]^ This ensures that the strain energy is absorbed by the interconnects during large deformation without significantly affecting the functional components in acquiring the biological signals.^[^ ^26^ ^]^ In addition, they serve as a connecting medium between flexible and rigid components such as sensors and printed circuit boards (PCBs) while providing shielding against environmental interferences like electromagnetic waves and temperature changes. This manifests that interconnects need not only high electrical conductivity and mechanical flexibility but also stable rigid‐soft connections for the device's integrity.

To meet those stringent requirements, many approaches have been widely researched and developed, as illustrated in Figure 1. One is the direct usage of intrinsically stretchable materials and engineered composite materials as interconnects. Stretchable materials such as liquid metals (LMs) and conductive polymers (CPs) are utilized in flexible substrates as they are mechanically durable and can achieve high electrical conductivity. Another approach involves devising sophisticated structures of traditionally rigid conductive materials to achieve stretchability.^[^ ^27^, ^28^ ^]^ For example, the most commonly used metal foil‐based interconnects cannot achieve the large deformation required in wearables due to their poor stretchability, which is typically less than 5%.^[^ ^29^ ^]^ By patterning into buckling structures, the metal interconnects can withstand stretching over 300%.^[^ ^30^ ^]^ To address the limitations of individual materials and harness their complementary advantages, the design of composite materials has emerged as a promising alternative approach for stretchable electronics. One widely used strategy is blending rigid conductive fillers into insulating polymer matrices to form conductive elastomers.^[^ ^31^ ^]^ Moreover, advanced materials and structural designs have been specifically engineered to function as rigid‐soft interconnects, mitigating the stress concentrations at the critical interface between disparate components. Approaches such as solid–liquid bilayer architectures, heterogeneous material integrations, and the incorporation of strain‐recoverable functionalities have been explored to establish robust interfacial connections.^[^ ^32^, ^33^ ^]^

In this review, we present recent advancements in stretchable interconnects for wearable bioelectronics by introducing various materials and structures to achieve electrically conductive functional components and mechanically compatible skin analogs concurrently. Different classes of materials are introduced and examined, regarding their inherent and tunable characteristics, current challenges, and promising potentials for viable stretchable interconnects. We then review compatible patterning strategies to achieve thin 2D and complex 3D circuits, as well as the corresponding input/output (I/O) technologies enabled by unconventional approaches for device integrity. Finally, we elucidate the challenges and opportunities in the development of stretchable interconnects and provide their prospects for future wearable health technologies.

## 2 Stretchable Conductive Materials and Structures

### 2.1 Stretchable Structural Design

**Figure 2:**
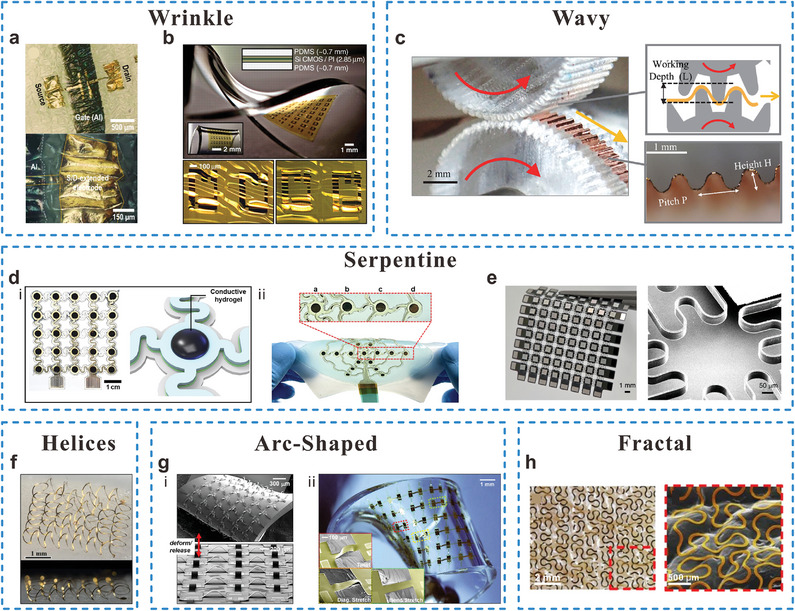
Structural designs for interconnects based on non‐stretchable materials. Wrinkled gold thin film on elastomer substrates as interconnects and source/ drain electrodes for a) stretchable organic transistors and b) stretchable and foldable integrated circuits (ICs) (Reproduced with permission.^[^ ^34^ ^]^ Copyright 2020, American Chemical Society. Reproduced with permission.^[^ ^35^ ^]^ Copyright 2008, American Association for the Advancement of Science), c) Copper film with wavy structure (Reproduced with permission.^[^ ^36^ ^]^ Copyright 2020, Engineering Reports published by John Wiley & Sons, Ltd.), Serpentine structured stretchable interconnects on d) multichannel sensor array and e) wearable light emitting diode array (Reproduced with permission.^[^ ^37^ ^]^ Copyright 2024, American Association for the Advancement of Science. Reproduced with permission.^[^ ^38^ ^]^ Copyright 2023, Springer Nature with a Creative Commons CC‐BY license), f) Gold interconnect with 3D helical structure (Reproduced with permission.^[^ ^39^ ^]^ Copyright 2017, Springer Nature with a Creative Commons CC‐BY license.), g) Arc‐shaped Au interconnect on stretchable ICs (Reproduced with permission.^[^ ^42^ ^]^ Copyright 2008, PNAS), and h) fractal‐inspired Peano‐based metal wires on the skin (Reproduced with permission.^[^ ^43^ ^]^ Copyright 2014, Springer Nature) are shown.

Conformal integration of wearable devices with the intricate surfaces of biological tissues often requires the devices to have a high degree of deformability while maintaining electronic performance. To preserve the performance of the functional components, the interconnects are usually designed to have stretchability by their relatively slender profile and large spatial occupancy. Many rigid or brittle materials, such as metals, exhibit excellent conductivity, making them prime candidates for high‐performance device interconnects. To enable stretchability, they are often patterned onto stretchable substrates in diverse shapes, including wrinkle,^[^ ^34^, ^35^ ^]^ wavy,^[^ ^36^ ^]^ serpentine,^[^ ^37^, ^38^ ^]^ helices,^[^ ^39^, ^40^ ^]^ arc‐shaped,^[^ ^41^, ^42^ ^]^ and fractal.^[^ ^43^ ^]^ as shown in **Figure** **2**. While these geometries can theoretically enhance the stretchability of interconnects regardless of the material, the electrical performance of rigid conductors and inherently stretchable conductors varies differently with strain. Typically, relatively brittle materials are fabricated or pre‐strained into thin and curved structures. During mechanical deformation, these structures can be stretched to return to a planar state, hence, achieving stretchability. The cross‐sectional area and length of the conductor itself remain mostly unchanged before and after stretching. Consequently, their resistance changes can be negligible within the tolerable strain range. Yet, a marginal increase in strain, for example, as little as 5% beyond the pre‐strain level, can result in a dramatic decline in device performance due to adhesive or cohesive failure by the generation of cracks, delamination, and disconnection of conductive metals.^[^ ^34^, ^44^ ^]^ In contrast, inherently stretchable conductors, such as LMs or ionogels, have lower moduli than the substrate, so substrate deformation can drive the conductive channel to deform as well.^[^ ^45^ ^]^ This results in larger resistance changes compared to brittle materials. Nevertheless, through structure design and conductive network engineering, the impact of this resistance variation on overall device performance can be mitigated. In such cases, the structural design aims more to compensate for the deformation of the conductive pathways during stretching, maintaining stable electrical performance, rather than enhancing stretchability.^[^ ^46^ ^]^

**Table** **1** summarizes and compares the performance of various interconnect structures and materials in stretchable devices. Well‐designed structures can ensure that the strain distribution on the conductive materials remains minimal even under large overall device stretching.^[^ ^41^, ^47^ ^]^ In comparison, 3D structures like helices exhibit even greater stretchability. However, they require a certain thickness determined by the desired strain, as the overall system strain is predominantly accommodated by changes in the amplitude perpendicular to the substrate due to buckling.

**Table 1:** Comparison table of stretchable interconnect structures and materials.

Structure	Conductive materials	Substrate	Stretchability	Resistance change	Patterning method	Application	Refs.
Wrinkle	Cu	Ecoflex	50%	Negligible (50% Strain)	Shadow Mask	Multifunctional Wearable Sensors	[55]
	Au/Al	Ecoflex	60%	Negligible (50% Strain)	Shadow Mask	Motion Sensor	[34]
	Graphene	Polydimethylsiloxane (PDMS)	80%	<5% (10% Strain)	Solution Coating	Stretchable Conductor	[56]
	Graphene	PDMS	100%	Negligible (80% Strain)	Lithography	Self‐Powered Tactile Sensor	[57]
Serpentine	Au	PDMS	100%	Negligible (100% Strain)	Lithography	Wearable Gas Sensors	[58]
	Au	Parylene‐C	350%	<2% (300% Strain)	Lithography	Curved Display	[38]
	Carbon Nanotube (CNT)	PDMS	1000%	Negligible (100% Strain)	Screen Printing	Energy Harvesting	[59]
	Multi‐Walled Carbon Nanotubes (MWCNT)	Waterborne Polyurethane (WPU)	100%	<1.6% (100% Strain)	Molding	Wearable Heater	[60]
	Graphene	PDMS	220%	Negligible (<20%)	Laser Printing	Multifunctional Wearable Sensors	[61]
Helices	Au	Ecoflex	≈120%	Negligible (50% Strain)	Lithography	Multifunctional Wearable Sensors	[39]
	Cu	Polyurethane (PU)/PDMS	150%	0.5% (100% Strain)	‐	Electrophysiological Sensors	[62]
	Cu	PU	500%	0.07% (200% Strain)	Pre‐Strain Release	Washable Electronics	[63]
	Polyaniline/MXene	PDMS	200%	25.4% (200% Strain)	Pre‐Strain Release	Conductive Yarn	[64]
	Poly(3,4‐ethylenedioxythiophene): Poly(styrenesulfonic acid)(PEDOT:PSS)	Elastic fiber	400%	Negligible (400% Strain)	Wet‐Spining	Stretchable Supercapacitors	[65]
Arc‐Shaped	Au	Polyimide (PI)	140%	Negligible	Lithography	Stretchable ICs	[42]
	Au	PI	10%	Negligible	Lithography	Curved ICs	[66]
Mesh‐Structure	Ni	PDMS	50%	<100% (50% strain)	Lithography	Transparent Heater	[67]
	Ag NWs	Thermoplastic Polyurethane (TPU)	≈100%	10.3% (50% strain)	Laser Printing	Wearable Heater	[27]
	Graphene	PDMS	20%	–	3D Printing	Temperature Sensor	[51]
Kirigami	Al	Ecoflex	643%	Negligible (500% Strain)	Electronic Cutting	Stretchable Heaters	[68]
	Eutectic Gallium Indium (EGaIn)	Silicon Rubber	820%	33% (820% Strain)	Laser Cutting	Tough Sensor and EEG Sensor	[49]
	Graphene	PI	240%	<0.25% (240%)	Reactive Ion Etching	Health Monitoring Devices	[69]

For materials that are difficult to pattern into curved geometries, stretchable structures can also be achieved through geometric design, such as mesh structures, origami,^[^ ^48^ ^]^ kirigami,^[^ ^49^ ^]^ or porous.^[^ ^50^ ^]^ structures. Mesh structures like honeycomb structures distribute strain through changes in the grid shape upon stretching.^[^ ^51^, ^52^ ^]^ These structures typically require both the substrate and the conductive components to conform to the same geometric design, often resulting in good breathability. Moreover, similar to kirigami, these regular linear geometric designs can only accommodate stretching in a few specific directions. Porous structures generally require a stretchable substrate, into which ordered or random voids are introduced and then covered or filled with conductive materials. These devices tend to be thicker, offering not only stretchability but also compressibility. Due to the volume changes of the internal cavities during stretching, they are often used for capacitive pressure sensors.^[^ ^53^ ^]^ However, when combined with fluidic conductive materials, the cavity changes can compensate for the deformation effects, enabling stretchability‐insensitive conductive interconnects.^[^ ^54^ ^]^

The suitability of different structures varies with material properties. For instance, due to the inherent discontinuity of CNT and graphene films, it is challenging to implement compressive strain‐induced buckling structures.^[^ ^70^ ^]^ Instead, the serpentine geometry is more commonly employed for stretchable conductors fabricated from these materials. Therefore, the role of each structure in the design will be discussed in more detail in the following sections, in conjunction with the relevant material characteristics.

### 2.2 Metallic Materials

Metals have predominantly been employed as interconnects in electronic devices due to their high electrical conductivity. Despite the inherent rigidity of bulky metals, mechanical flexibility can be achieved by reducing their thickness to nanometer scale.^[^ ^71^ ^]^ The stretchability of metal films is further introduced by decorating them in rational fractal designs to withstand strain through geometric structures. In parallel, metal fillers can be added to the stretchable materials to make composites for the formation of micro/nanostructure percolation networks such that the elastomer matrix absorbs the strain energy while preventing the breakage of conductive metal pathways. In this section, we present an overview of stretchable metal‐based interconnects, focusing on the underlying mechanisms, methodologies, and potential applications in wearable bioelectronics.

#### 2.2.1 Structured Metal Thin Film

**Figure 3:**
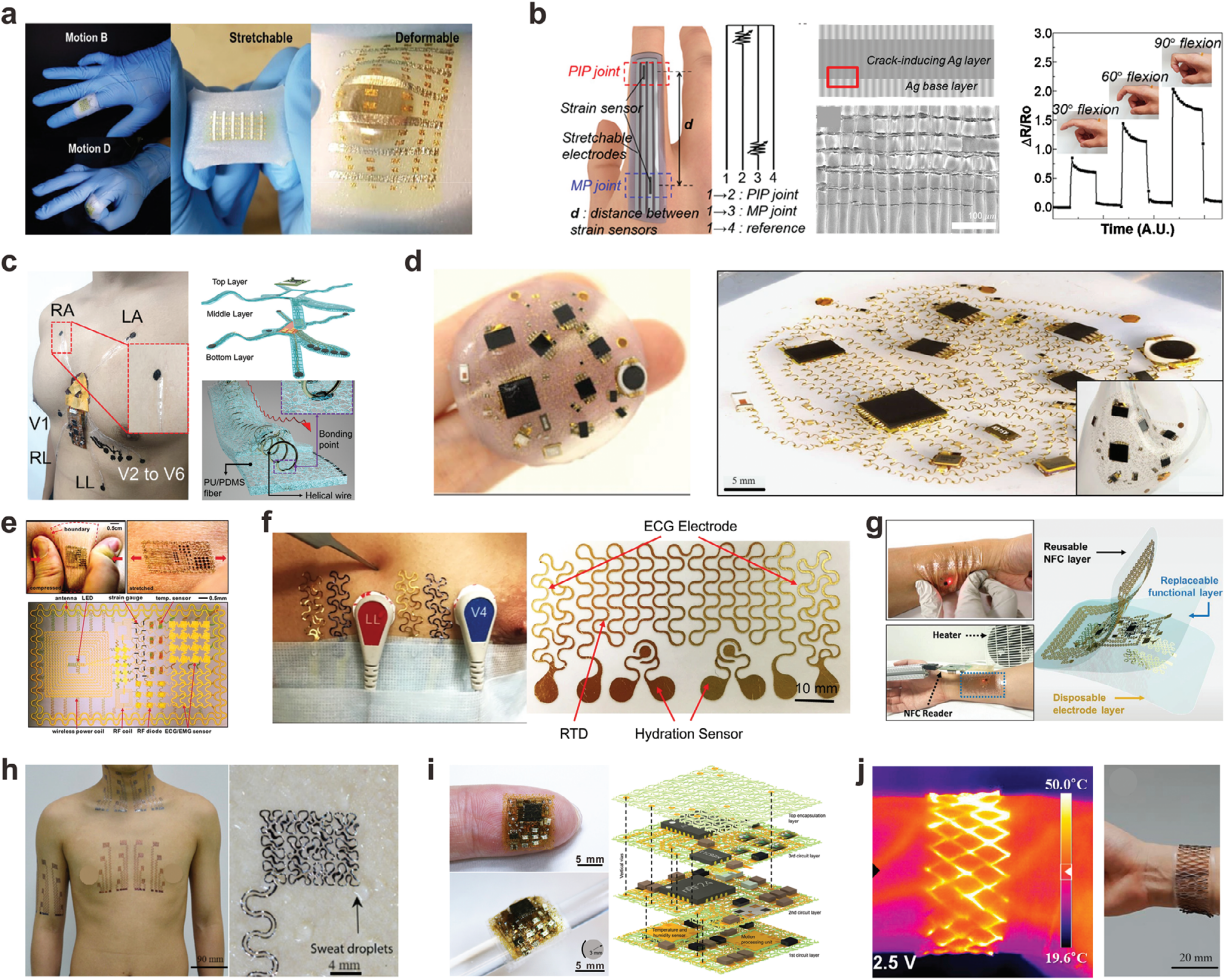
Structured metal interconnects integrated on stretchable wearable bioelectronics. a) Wearable motion sensor with wrinkle interconnects (Reproduced with permission.^[^ ^34^ ^]^ Copyright 2020, American Chemical Society), b) Wearable strain sensor with wrinkle Ag interconnects (Reproduced with permission.^[^ ^73^ ^]^ Copyright 2020, Elsevier B.V.), c) 12‐lead ECG patch with epidermal 3D helical interconnects (Reproduced with permission.^[^ ^62^ ^]^ Copyright 2023, Wiley‐VCH GmbH), d) Soft sensor with self‐assembled 3D interconnects (Reproduced with permission.^[^ ^39^ ^]^ Copyright 2017, Springer Nature with a Creative Commons CC‐BY license), e) E‐tattoo with serpentine interconnects (Reproduced with permission.^[^ ^77^ ^]^ Copyright 2011, American Association for the Advancement of Science), f) E‐tattoo ECG sensor with serpentine interconnects (Reproduced with permission.^[^ ^78^ ^]^ Copyright 2018, Springer Nature with a Creative Commons CC‐BY license), g) Modular and reconfigurable E‐tattoo sensor (Reproduced with permission.^[^ ^22^ ^]^ Copyright 2019, WILEY‐VCH), h) 16 channel ECG and sEMG sensor with serpentine and fractal interconnects (Reproduced with permission.^[^ ^79^ ^]^ Copyright 2020, American Association for the Advancement of Science), i) Highly integrated stretchable electronic system (Reproduced with permission.^[^ ^80^ ^]^ Copyright 2022, American Association for the Advancement of Science), and j) Stretchable heater based on Kirigami patterning (Reproduced with permission.^[^ ^68^ ^]^ Copyright 2017, American Chemical Society) are displayed.

Structural metal films are developed by patterning metals in a special design such that the design can withstand geometrical shape changes.^[^ ^28^ ^]^ One of the most straightforward structural designs employed for stretchable metal interconnects is the wrinkled geometry, which can be fabricated in a simple and cost‐effective manner at large scales. Specifically, a wrinkled structure is prepared by inducing strains in a substrate on which a thin metal film is deposited, causing the metal to buckle and form regular or irregular wrinkles when the strain is released. The presence of the wrinkle structure effectively relieves the internal stress that otherwise will arise in the metals, mitigating the potential electrical failure by disconnection of the conductive pathways. The mechanics, geometries, and fabrication methods for wrinkled patterns have been reviewed in detail by Lee et al.^[^ ^72^ ^]^ The use of biaxially‐stretched substrates enabled the fabrication of omnidirectional stretchable Au and Al interconnects that demonstrated stable electrical performance under 60% strain over 10 000cycles, making them suitable for human motion monitoring applications (**Figure** **3**a).^[^ ^34^ ^]^ Similarly, wrinkled Ag interconnects designed for finger flexion sensing exhibited negligible resistance change under 50% tensile strain for 1000 cycles, matching the deformability of human skin (Figure 3b).^[^ ^73^ ^]^ However, the stretchability of the wrinkled structures is limited by the pre‐strain applied during fabrication, which constrains the stability and performance of these devices within that strain threshold.

Alternatively, wavy patterns, 3D helices, and arc‐shaped configurations can be also developed on pre‐stretched substrates.^[^ ^74^, ^75^ ^]^ These structures offer more advantages over wrinkled structures because they allow out‐of‐plane deformation in the controlled region, offering higher stretchability and consistency. A recent work by Li et al. demonstrated a 3D helical copper interconnect embedded in a soft PU/PDMS shell, inspired by accordion lanterns.^[^ ^62^ ^]^ This interconnect exhibited a maximum strain of 150% with only a 0.5% relative resistance change under 100% strain, and showed exceptional electrical stability with a high signal‐to‐noise ratio during dynamic deformation, making it suitable for applications like flexion and heart activity monitoring (Figure 3c). Similarly, Jang et al. transformed a 2D mesh‐type serpentine structure into a 3D helical coil by compressive buckling on a pre‐stretched Ecoflex substrate, as shown in Figure 3d. The integrated device with these helical interconnects and rigid IC chips could accurately monitor various electrophysiological signals like ECG and EEG even when stretched up to 50% strain. These examples showcase the advantages of 3D structural interconnects in enhancing the stretchability beyond the limits of the supporting substrates. Consequently, the failure of such interconnect‐based devices is primarily attributed to the stretching limit of the substrate rather than the metal wires themselves.

Instead of deforming the substrates or shaping the metal components into relatively thick 3D structures, metals can be configured into sophisticated 2D patterns on flat, non‐strained substrates to achieve multi‐directional stretchability. Planar structures like serpentine, fractal, and spiral patterns are widely used for the development of ultrathin stretchable devices. For example, serpentine‐shaped interconnects are commonly used for electronic tattoos (e‐tattoos) and on‐skin electronics, as shown in Figure 3e–h.^[^ ^76^ ^]^ In 2011, the Rogers group reported the first e‐tattoo capable of conforming to the microscale surface textures of human skin by serpentine interconnects based on water‐soluble PVA thin film.^[^ ^77^ ^]^ E‐tattoos offer advantages such as compliance, breathability, and seamless integration with the skin, leading to minimal motion artifacts and negligible susceptibility to perspiration.^[^ ^78^ ^]^ By incorporating various electronic elements and physical sensors, e‐tattoos can be further developed into modular and reconfigurable multifunctional wearable systems.^[^ ^22^ ^]^ The design of double‐stranded serpentine antennas and single‐stranded interconnects ensures mechanical and electrical stability under up to 20% skin‐tolerable strains. Nevertheless, the delicate and fragile nature of e‐tattoos poses challenges in achieving stable alignment and large‐area placement on the skin. To address this, a novel approach using a sphere rolling method was demonstrated, enabling a filamentary serpentine e‐tattoo to achieve 560% stretchability in the freestanding state and successfully capture high‐fidelity 16‐channel surface EMG and ECG signals.^[^ ^79^ ^]^

Planar interconnects can overcome spatial constraints by penetrating 3D substrates. Researchers combined serpentine interconnects with fractal‐structured PI substrates, mimicking the mechanical response of human skin (Figure 3i).^[^ ^80^ ^]^ This allowed the serpentine interconnects to undergo lateral buckling and out‐of‐plane deformations to release strain energy. The resulting multilayer device system exhibited remarkable robustness, withstanding 20% stretching, 15% pressing, 30‐degree shearing, and 540‐degree twisting. Despite its small size, the device achieved high functional density, functioning as a compass, skin temperature/humidity sensor, motion and heart rate monitor, and somatosensory mouse. While highly integrated devices limit the available space for serpentine or fractal structures, incorporating 2D spiral structures can offer higher levels of elongation than serpentine interconnects.^[^ ^28^ ^]^ Spiral interconnects with a uniform and small curvature, such as the Archimedean spiral, are reported to possess greater stretchability compared to the golden spiral.^[^ ^81^ ^]^ However, the requirement for rotation to unfurl the spiral structure imposes constraints on patterning strategies and application scenarios.^[^ ^82^, ^83^ ^]^

In addition to curved structures, non‐stretchable conductive layers can be patterned with kirigami and fabric to accommodate extreme deformations.^[^ ^84^, ^85^ ^]^ Kirigami‐inspired microcracked structures endowed MXene sheets with highly stable conductivity even when stretched to 100%, enabling wearable touch sensors.^[^ ^86^ ^]^ Selective metal deposition on kirigami substrates can create interconnects with both resistance‐independent and resistance‐sensitive behavior, which can be also controlled by adjusting the cutting length.^[^ ^87^, ^88^ ^]^ The kirigami approach has also enabled stretchable and flexible wearable heaters using aluminum paper as shown in Figure 3j. These heaters can maintain intimate conformal contact with curvilinear body surfaces and demonstrate durability even after 1000 cycles at 300% strain.^[^ ^68^ ^]^ Natural textile materials like cotton, wool, and silk can also serve as device substrates, allowing metal‐coated fibers to be seamlessly integrated into clothing for unobtrusive health monitoring.^[^ ^89^, ^90^ ^]^

Metal thin films are undoubtedly the most widely used electronic interconnects due to their stability and compatibility with various patterning approaches. However, their thin nature introduces sheet resistance challenges and susceptibility to damage from abrasion. Coupling thin metal layers with soft polymer substrates also prevents effective soldering due to thermal limitations. This necessitates the development of softer electronic components, packaging technologies, and advanced connection methods to fully utilize metallic interconnects in real‐world health monitoring applications. Numerous soft I/O connectors have been developed to address these challenges, which will be discussed in the “I/O connectors” section.

#### 2.2.2 Metal Composites

**Figure 4:**
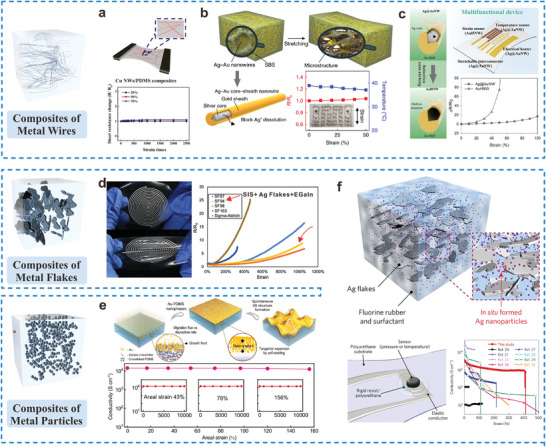
Strain‐invariant percolation networks formed by metal fillers in polymer matrices. a) Composite of Cu NWs and PDMS with stable resistance under stretching (Reproduced with permission.^[^ ^91^ ^]^ Copyright 2022, Elsevier B.V.), b) Ag–Au NWs in poly(styrene‐butadiene‐styrene) (SBS) with stable resistance under strain (Reproduced with permission.^[^ ^93^ ^]^ Copyright 2018, Springer Nature with a Creative Commons CC‐BY license), c) Multifunctional device with Ag–Au NWs and Au hollow NWs (Reproduced with permission.^[^ ^94^ ^]^ Copyright 2023, Wiley‐VCH.), d) Composite of SIS, EGaIn, and Ag flakes with low resistance change under stretching (Reproduced with permission.^[^ ^98^ ^]^ Copyright 2022, Wiley‐VCH), e) Metal‐elastomer nanophases with highly strain‐invariant electrical conductivity (Reproduced with permission.^[^ ^106^ ^]^ Copyright 2024, Springer Nature with a Creative Commons CC‐BY license), and f) Composite containing Ag flakes and NPs as a stable interconnect of a wearable sensor (Reproduced with permission.^[^ ^108^ ^]^ Copyright 2017, Springer Nature) are presented.

Composite materials present a feasible alternative to conventional nonstretchable and easy‐disintegrable metallic interconnects. By integrating micro/nano metallic materials, metal oxide wires, flakes, and particles into stretchable materials, interconnects that are both mechanically stretchable and electrically conductive can be attained. Incorporation of nanomaterials such as nanowires (NWs) is advantageous as their high aspect ratios provide percolated paths for electrical conduction even at high strains. For example, Cu NWs spray‐coated on a wrinkled PDMS substrate demonstrated a stable sheet resistance under 2500 cycles at a 70% strain (**Figure** **4**a).^[^ ^91^ ^]^ A stretch‐insensitive capacitive pressure sensor based on this interconnect demonstrated less than 0.8% change in capacitance when it was stretched below 200% strain. The use of NWs that are longer than 100 µm can further extend the percolated paths, and hence, reduce the resistance change due to inter‐NW junction breakage, simultaneously achieving good conductivity and stretchability.^[^ ^92^ ^]^ To eliminate the potential health issue associated with the use of Ag NWs, Choi et al. developed an Ag–Au core–shell nanocomposite by coating a Au layer onto Ag NWs (Figure 4b).^[^ ^93^ ^]^ This nanocomposite exhibited a conductivity exceeding 7 × 10^4^ S cm^−1^ and a stretchability of up to 840%. Furthermore, the core‐shell nanocomposites can be transformed into hollow NWs as shown in Figure 4c, via a controlled etching process, to enable more versatile electrical properties for usage as strain sensors, heating systems, and strain‐gated circuits in wearable devices.^[^ ^94^ ^]^ This advancement paves the way for the development of intelligent wearable devices that incorporate monolithically patterned Au NWs, capable of performing signal monitoring and processing functions simultaneously.

Alternatively, metallic flakes and nanoparticles (NPs) composites provide a facile method for fabricating interconnects that are insensitive to strain, owing to their self‐organization capabilities during stretching.^[^ ^95^ ^]^ Due to the small size of fillers, these metal‐elastomer composites can be easily prepared through printing and spraying.^[^ ^96^, ^97^ ^]^ For example, a biphasic ink formulation consisting of Ag microflakes, EGaIn alloy, and styrene isoprene (SIS) was developed to be compatible with affordable extrusion printers (Figure 4d).^[^ ^98^ ^]^ The negatively charged Ag was absorbed on the positively charged EGaIn to enhance wettability and conductive pathways in the SIS binder. By controlling the Ag microflake density, the ink achieved a high conductivity of 6.38 × 10^5^ S m^−1^ and an excellent stretchability of over 1000%. Utilizing a soft elastomeric SIS and a low‐temperature fabrication process, the composite ink served as stretchable and stable interconnects on wearable ECG sensors. Metallic NPs exhibit better compatibility with numerous patterning technologies such as screen printing,^[^ ^99^ ^]^ electrospinning,^[^ ^100^ ^]^ aqueous self‐assembly,^[^ ^101^, ^102^, ^103^ ^]^ and lithography.^[^ ^104^ ^]^ However, composite materials with NP fillers typically exhibit lower conductivities due to their lower aspect ratios compared to flake‐ or wire‐shaped fillers. The separation of particles during stretching can significantly increase the resistance. Therefore, a high loading of fillers is required, which in turn compromises the mechanical stretchability.^[^ ^105^ ^]^ To concurrently preserve stretchability and conductivity, Chae et al. recently proposed a thin‐film evaporation approach that leverages fluxes of the PDMS crosslinkers and Au atoms in a PDMS substrate (Figure 4e).^[^ ^106^ ^]^ The controlled migration flux of excessive crosslinkers from PDMS upon the introduction of Au NP on PDMS facilitated the spontaneous formation of an Au‐PDMS nanophase. The presence of reticular nanophases with gyrification and tangential stress gradients enabled the film to exhibit stretchability similar to elastomers while maintaining metal‐like conductivity (1.4 × 10^4^ S cm^−1^). These materials demonstrate consistent conductivity even after 10 000 stretching–unstretching cycles and good durability under harsh conditions. Likewise, Liu et al. devised a material with exceptional stretchability and stability through the deposition of a wrinkled Au film on a semi‐polymerized PDMS, leveraging the interlocking effect between the metal particles and the polymer substrate.^[^ ^107^ ^]^ The resulting skin‐like conductor showed over 130% stretchability and maintained electrical stability under cyclic stains (>10 000 cycles), showing good performances as EMG and motion sensors.

Furthermore, the conductivity of particle‐based interconnects can be strengthened by forming percolated networks with high‐aspect‐ratio fillers. As shown in Figure 4f, uniformly dispersing Ag NPs along with Ag microflakes created an optimized percolated network, where electron tunneling between Ag NPs significantly enhanced carrier transport, resulting in over 1000 S cm^−1^ conductivity even under 400% strain.^[^ ^108^, ^109^ ^]^

The resistance of metal composites depends on the inherent conductivity of nanomaterials and the junction resistance between fillers. Although these composites can exhibit high conductivity and mechanical stability concurrently, fracture of filler‐to‐filler junctions upon deformation can lead to a significant rise in sheet resistance. Potential strategies like dense networks, filler alignment, and interfacial energy modulation offer promising ways to further refine the properties of metal composite interconnects.^[^ ^105^, ^110^, ^111^ ^]^

#### 2.2.3 Liquid Metals

LMs refer to metals or their alloys which exist in a stable liquid state or melt at or near room temperature. They possess low melting points, such as the commonly‐known mercury (−38.8 °C), cesium (28.5 °C), and gallium (29.76 °C). Under the consideration of toxicity and chemical reactivity, Ga and its alloys have been the most widely‐utilized LMs among the existing counterparts. Ga‐based LMs have low vapor pressures and non‐toxicity. Additionally, they are suitable for flexible electronics due to low viscosity, excellent fluidity, and high electrical and thermal conductivity. They have been exploited in diverse biomedical applications, including drug delivery,^[^ ^112^ ^]^ enhanced cancer therapy,^[^ ^113^, ^114^, ^115^ ^]^ molecular imaging,^[^ ^113^ ^]^ ultrasound imager,^[^ ^116^ ^]^ and flexible bioelectronics.^[^ ^116^, ^117^ ^]^ Notably, EGaIn and eutectic Ga–In–Sn alloy (Galinstan) are the two most commonly used and commercially available Ga‐based LMs. These alloys have lower melting points (15.7 and 11 °C for EGaIn and Galinstan, respectively) than Ga. Additionally, they exhibit exceptionally high electrical conductivity which can reach up to 10^6^ S m^−1^, which is far superior to ordinary liquids.^[^ ^117^, ^118^, ^119^ ^]^ These attributes make them very attractive for various applications such as conductive electrodes, antennas, and interconnects within the realm of stretchable bioelectronics.^[^ ^120^, ^121^, ^122^ ^]^

**Figure 5:**
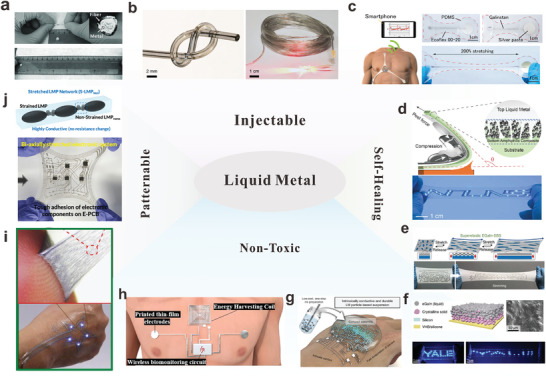
LM‐based stretchable interconnects. a) Ultra‐stretchable high conductive fibers formed by injecting LM into hollow elastomer (Reproduced with permission.^[^ ^123^ ^]^ Copyright 2013, WILEY‐VCH), b) Soft and stretchable  LM channels in SEBS (Reproduced with permission.^[^ ^124^ ^]^ Copyright 2020, Springer Nature), c) Transformable ECG patch with LM interconnects (Reproduced with permission.^[^ ^33^ ^]^ Copyright 2021, American Chemical Society), d) Ultrahigh strain‐insensitive electronics using LM conductor (Reproduced with permission.^[^ ^54^ ^]^ Copyright 2022, Wiley‐VCH), e) Permeable and stretchable LM fiber mat (Reproduced with permission.^[^ ^126^ ^]^ Copyright 2021, Springer Nature), f) Highly stretchable interconnects formed by biphasic GaIn (Reproduced with permission.^[^ ^32^ ^]^ Copyright 2021, Springer Nature), g) LM‐based e‐tattoo bioelectronics (Reproduced with permission.^[^ ^127^ ^]^ Copyright 2022, Wiley‐VCH), h) Skin electronics with LM interconnects (Reproduced with permission.^[^ ^128^ ^]^ Copyright 2020, Springer Nature with a Creative Commons CC‐BY license), i) LM‐based skin electronics without residues after peeling (Reproduced with permission.^[^ ^129^ ^]^ Copyright 2022, Wiley‐VCH), and j) Elastic PCB with LM polymer composite as the interconnects (Reproduced with permission.^[^ ^130^ ^]^ Copyright 2022, American Association for the Advancement of Science) are shown.

Due to their liquid states, LMs are expected to have infinite stretchability. Therefore, LMs can be injected into predefined flexible structures (**Figure** **5**a,b) to achieve highly conductive and stretchable interconnects without disintegration concerns as seen in solid metals‐based interconnects.^[^ ^24^, ^123^, ^124^ ^]^ However, the fabrication of elongated and narrow LMs channels in soft and stretchable materials poses a technical challenge, limiting the choice of substrate materials and hindering 3D integration. To overcome this limitation, researchers have explored the plasticity of solid‐state GaIn alloy to fabricate 3D structured LM electronics.^[^ ^125^ ^]^ Instead of fabricating channels on the substrate, a solid alloy of Ga and 10% weight In was cooled to form solid metallic complex circuits. The circuits were then encapsulated in elastomers and heated (at 22.7 °C) to regain fluidity, allowing the connection of other electronic components when the interconnects return to a liquid state. Moreover, LMs can maintain their fluidity over a wide range of temperatures due to the supercooling effect, enabling the embedding of LM circuits in substrates such as Ecoflex without the need for bonding processes. The final device demonstrated excellent stretchability and circuit stability in a wearable finger motion monitoring device.

LM can easily flow and deform along with the geometrical shape changes of the microfluidic channels, ensuring electrical stabilities even under large deformation. Ultrathin LMs‐based microfluidic channels have been used in highly sensitive strain sensors.^[^ ^45^, ^131^ ^]^ Despite the relatively low resistance changes exhibited by LM interconnects during deformation when compared to many physiological sensing systems (Figure 5c), there is a possibility of interference with the final signal, leading to intrinsic noise and potential failure of detection in certain movement detection systems.^[^ ^33^, ^54^, ^132^ ^]^ Leveraging the fluidity and self‐healing properties of LMs, structured LM films can maintain stable electrical characteristics during stretching due to volume compensation.^[^ ^133^, ^134^ ^]^ For example, a highly stretchable (2260%) LM‐based interconnect was developed by creating a liquid‐solid bilayer.^[^ ^54^ ^]^ Researchers fabricated a solid composite containing LM particles (LMP) in polyester polyol‐rich TPU. These LMP‐TPU composites can be stencil‐printed into smooth and uniform patterns on different polymer substrates. Upon peeling the patterns off from the substrate, the LMPs in the composites form a dense LM layer on the surface, resulting in a bilayer liquid‐solid composite as shown in Figure 5d. The bilayer structure allowed the LM to self‐organize and maintain a continuous conductive path, even as the LMP‐TPU composite ruptured. This compensating effect limited the resistance change to 30%, which was lower than the bulk metal as predicted by Pouillet's law. This strain‐resilient layout demonstrated promising potential in the applications of wearable tactile sensors and heaters. Similarly, the LMs can be transformed into mesh‐like structures hanging on the electrospun polymer microfiber mat (Figure 5e).^[^ ^126^ ^]^ The LM fiber mat could retain ultrahigh conductivity (up to 1.8  × 10^6^ S m^−1^) with negligible resistance loss for over 1800% stain. These strain‐insensitive properties are attributed to the reversible structure change of LM in the polymer matrix, facilitated by the formation of a thin solid Ga oxide layer upon exposure to air.^[^ ^135^ ^]^ Alternatively, a biphasic layer can be generated by modulating the solid oxide formation by thermal sintering of LMs. As presented in Figure 5f, the thermal treatment of EGaIn NPs enabled a thin solid oxide film formation on the surface by oxidation and phase segregation.^[^ ^32^ ^]^ The solid phase allows better interfacial adhesion with rigid components, while the liquid phase ensures electrical connection under large strains. This biphasic LM conductor exhibited a stretchability of over 1000%, a conductivity of 2.06 × 10^4^ S cm^−1^, and a negligible resistance change upon straining. Due to their durability, these biphasic approaches offer the benefits of lower artifacts, enabling various physiological health monitoring applications. (Figure 5c,g,h).^[^ ^127^ ^]^

Despite their apparent advantages in developing wearables, LMs have shortcomings of uncontrollable and unstable morphology due to their low viscosity and high surface tension. This creates a potential failure of the devices. To tackle this issue, diverse strategies for anchoring LMs were developed.^[^ ^136^ ^]^ One approach is to utilize Ag epoxy as docking points for GaIn, as In can provide strong adhesion to Ag microparticles.^[^ ^128^ ^]^ Another method uses polyvinyl alcohol (PVA) as an adhesion medium between the skin and LMs for wear‐resistant epidermal electronics.^[^ ^137^ ^]^ PVA forms hydrogen bonds with LM oxides and adheres to the keratin in the epidermis, preserving the LM pattern even under abrasion. When directly patterned on the skin by airbrushing, this LM circuit maintained electrical stability under 800% strain and formed a ultra‐thin, conformal, and permeable micropattern. Achieving a strong adhesion between the LMs and their hosting materials not only ensures integrity of the device during deformation but also assists residual‐free removal of LMs upon peeling off from the skin. For instance, dispersing LM particles in acrylate polymers creates “sticky conductors” that can reliably attach to soft or rigid conductors, with the adhesion force and LM particle size adjusted to prevent residues during peeling (Figure 5i).^[^ ^129^ ^]^ Owing to the strong adhesiveness of the acrylates, the printed stretchable interconnects can attach onto soft or rigid conductors, enabling reliable interface connections.

Another major drawback of LM‐based electronics is the potential leakage upon rupture of LM structures, which hinders the utilization of these highly conductive and stretchable materials in practical wearable applications.^[^ ^138^ ^]^ Traditional approaches to pattern highly‐conductive LM composites by sintering can easily disrupt the LM oxides, hence leading to leakage of LMs.^[^ ^119^ ^]^ To address this challenge, a non‐destructive approach was developed to create a nearly defect‐free, long‐range network of large and small LMPs embedded in PU.^[^ ^130^ ^]^ By applying the acoustic energy to the LMP‐PU composites, nanometer‐scale cavities were generated in situ to form small LMPs connecting the larger ones. The large LMPs can deform as the network was strained while the small LMPs remain in place, maintaining a connective path between the large LMPs (Figure 5j). Due to the small size of LMPs network and the toughness of the LMP‐PU composites, no apparent leakage was observed under abrasion and tensile stress. This LM network exhibited stretchable and electrically stable interconnects for wearable photoplethysmography sensing. Furthermore, this strategy is universal to various polymer matrices, including photoresists such as SU‐8, enabling high‐resolution interconnect patterning.

As evident from the preceding examples, LMs have exceptional properties that cannot be achieved by traditional metals, making them an attractive material for flexible and stretchable bioelectronics. Their innate stretchability which can conform to their encasing substrates and high conductivity enables super‐stretchable interconnects.^[^ ^61^, ^139^, ^140^, ^141^ ^]^ Yet patterning LMs often involves manual steps such as sealing and injection and sophisticated procedures, making their manufacturing tedious and labor‐intensive.^[^ ^142^ ^]^ Furthermore, the utilization of Ga in LM systems incurs high costs, which could potentially be mitigated by leveraging microfabrication approaches where the materials’ demand is less. There has been research on tackling this limitation,^[^ ^143^ ^]^ and further details on micropatterning will be discussed in the “patterning method” section.

### 2.3 Polymer Materials

#### 2.3.1 Conductive Polymers

Polymers possessing both intrinsic conductivity and stretchability, such as polyacetylene, polyaniline, polypyrrole, and PEDOT, hold great promise for applications in stretchable bioelectronics. The stretchability of these polymers originates from their disordered molecular chains which can interchange between folded and unfolded states during deformation. CPs typically contain π‐conjugation in which delocalized π‐electrons can move along the alternating single and double bonds within the polymer skeleton.^[^ ^144^ ^]^ However, the low crystallinity of the CPs hampers the π‐electrons delocalization and retards charge transport, leading to weak charge conduction.^[^ ^21^ ^]^ Introducing electrical carriers through doping is a common method to enhance the conductivity of intrinsic conjugated polymers to 10 or more orders of magnitude.^[^ ^145^, ^146^ ^]^ While doping can lead to the formation of a metallic state in π‐conjugated systems, it also reduces structural and morphological disorder, making the material prone to cracking during stretching.^[^ ^147^ ^]^ Therefore, achieving concurrent improvements in conductivity and stretchability remains a significant challenge, limiting the application of CPs as stretchable interconnects. In light of recent advancements in stretchable wearable bioelectronics, highly conductive CPs that exhibit similar mechanical behavior as human skin have attracted research investigations.^[^ ^148^, ^149^ ^]^

**Figure 6:**
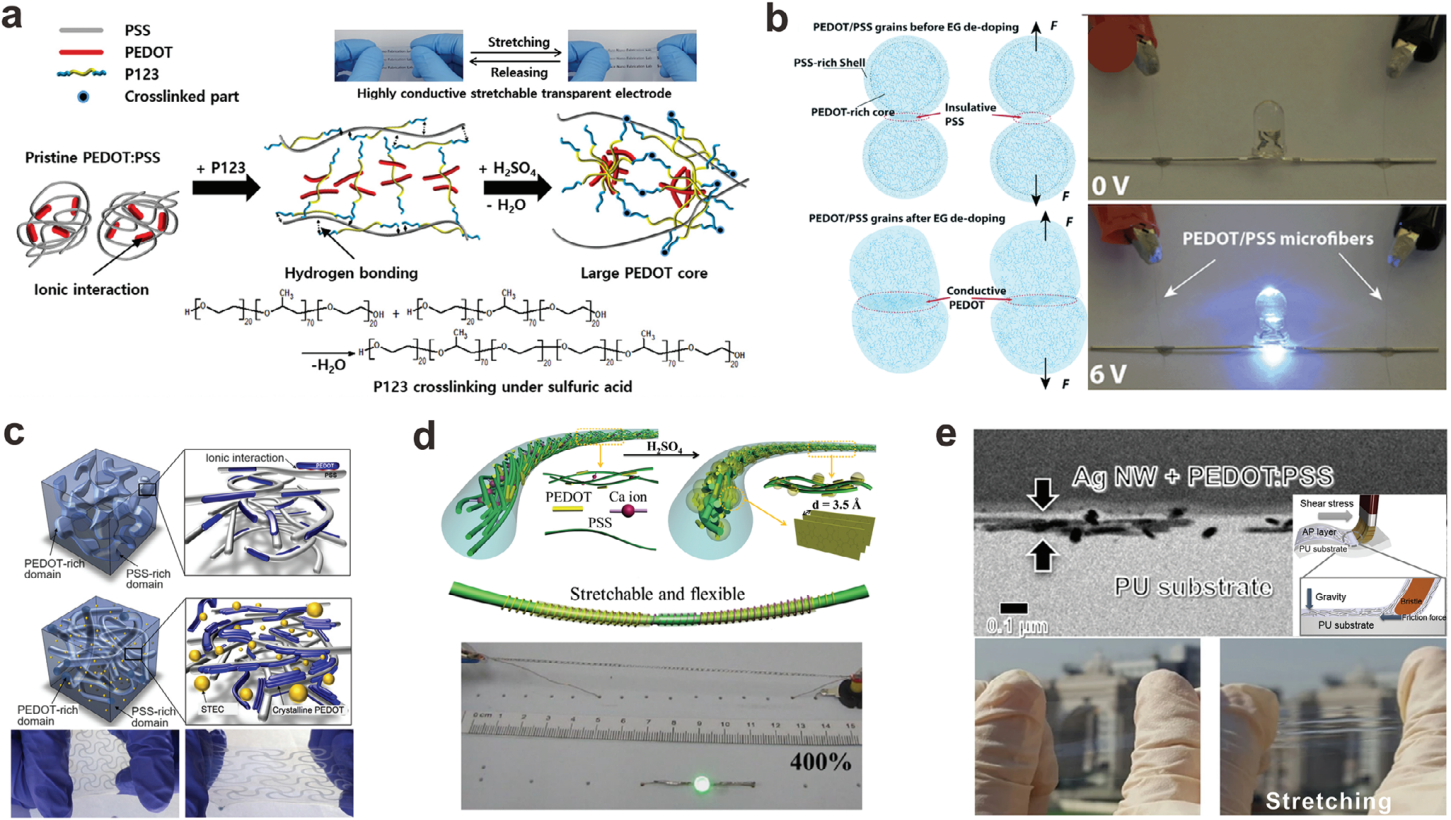
Highly conductive polymer‐based stretchable materials. a) Stretchable and transparent PEDOT:PSS‐based electrodes (Reproduced with permission.^[^ ^161^ ^]^ Copyright 2018, American Chemical Society), b) EG doped PEDOT:PSS conductive fibers (Reproduced with permission.^[^ ^162^ ^]^ Copyright 2015, Royal Society of Chemistry), c) A CP interconnect with highly stretchable and conductivity enhanced by STEC (Reproduced with permission.^[^ ^23^ ^]^ Copyright 2017, American Association for the Advancement of Science), d) Fiber interconnect and supercapacitors formed by PEDOT:PSS (Reproduced with permission.^[^ ^65^ ^]^ Copyright 2018, Elsevier), e) Highly stretchable Ag NWs/PEDOT:PSS composite electrode (Reproduced with permission.^[^ ^164^ ^]^ Copyright 2017, Springer Nature with a Creative Commons CC‐BY license) are exhibited.

Doping of the CPs is achieved through oxidation‐reduction reactions.^[^ ^146^ ^]^ For example, the electrical conductivity of PEDOT can be enhanced through the oxidation of PEDOT using anionic polyelectrolytes like PSS, which incorporates deprotonated sulfonyl groups. Additionally, the hydrophobic PEDOT chains can be stabilized by the hydrophilic PSSH chains, facilitating the formation of PEDOT:PSS through simple solution mixing processes. Despite the processing simplicity, pure PEDOT:PSS thin films exhibit low fracture strains (5–8%) due to the rigid PEDOT chains.^[^ ^150^, ^151^ ^]^ To extend the stretchability of PEDOT:PSS, various polymers such as poly(ethylene glycol),^[^ ^152^ ^]^ poly(vinyl alcohol),^[^ ^153^ ^]^ PDMS,^[^ ^154^ ^]^ natural rubber,^[^ ^155^ ^]^ and WPU.^[^ ^156^ ^]^ as well as plasticizers like  Zonyl,^[^ ^157^ ^]^ Triton X‐100,^[^ ^158^ ^]^ D‐sorbitol,^[^ ^159^ ^]^ and ionic liquid^[^ ^23^, ^160^ ^]^ have been blended or secondarily doped into PEDOT:PSS. For instance, the stretchability of PEDOT:PSS could be enhanced by mixing with an amphiphilic triblock poly(ethylene glycol)‐block‐poly(propylene glycol)‐block‐poly(ethylene glycol) that is commercially known as poloxamer (**Figure** **6**a).^[^ ^161^ ^]^ When this block copolymer‐incorporated PEDOT:PSS was treated with sulfuric acid, morphological and PEDOT:PSS ratio changes facilitate the electrical conductivity. Consequently, a conductivity of 1700 S cm^−1^ was achieved with slight changes in the conductivity under repetitive stretch–release cycles at 40% tensile strain. The polymer also depicted a lower Young's modulus than pristine PEDOT:PSS, bringing them closer to human skin's mechanical characteristics. The improved mechanical stretchability of PEDOT:PSS fibers has also been demonstrated by doping them with ethylene glycol (EG) (Figure 6b).^[^ ^162^ ^]^ The fabricated fibers exhibited an electrical conductivity of 2804 S cm^−1^ and a maximum strain of 21%. Instead of introducing stretchable polymers as doping elements to minimize phase separation between the conductive PEDOT domains, the addition of small plasticizers also allows both conductivity and stretch stretchability.^[^ ^163^ ^]^ One example is ionic additives–assisted stretchability and electrical conductivity enhancers (STEC) such as dioctyl sulfosuccinate sodium salt.^[^ ^23^ ^]^ The incorporation of STEC into PEDOT:PSS increases the crystallinity and interconnectivity of the PEDOT phase as STEC resides in a more disordered region of the CP. This simultaneously softens the PSS domains for better stretchability. As a result, the PEDOT:PSS/STEC interconnect exhibited a high conductivity of  4100 S cm^−1^ with 100% stretchability, as shown in Figure 6c. The utilization of a 3D helical structure for the sulfuric acid‐treated PEDOT:PSS fibers serves as an effective approach to enhance the stretchability of the fiber interconnects (Figure 6d). The configuration allows the interconnect and supercapacitor device to increase the stretchability from 23% to an impressive 400%, without compromising the high conductivity (1770 S cm^−1^).

Compared to structured metallic interconnects, the stretchable CP‐based interconnects can withstand similar strain at a smaller area, hence, they can be miniaturized to a smaller extent. Due to the ease of processing by solution‐based printing, CPs can easily be patterned at a microscale, providing design versatility in integrated stretchable wearable systems. However, the inherent limitations in electrical conductivity and the restricted material selection range of CPs often necessitate hybridization with other conductive materials to achieve desired electrical and mechanical performance characteristics.

#### 2.3.2 Conductive Polymer Composites

Stretchable conductive composites typically contain polymer matrices, which possess inherent softness and mechanical deformability, embedded with fillers with exceptional intrinsic electrical properties. The tunable physical properties and diverse functionalities render them highly appealing materials. Their high conductivities are achieved by the percolated network of highly conductive fillers, in which their types, concentrations, and geometries determine the achievable conductivity.^[^ ^165^, ^166^ ^]^ Morphologies of conductive fillers include particles (e.g., carbon black and metal particles), wires (e.g., CNT, metal NWs), and metal/ CP fibers), flakes (e.g., graphene, metal flake, MXene), and intrinsically‐deformable materials (e.g., LMs and CPs). In terms of substrates, elastic polymers such as PDMS, Ecoflex, SEBS, TPU, and WPU are most commonly used for developing wearable bioelectronics due to their low cost, facile processability, and excellent biocompatibility.^[^ ^167^, ^168^ ^]^ Since the metal‐polymer and carbon‐polymer composites are discussed in the corresponding sections, this section will briefly introduce the conductive composites based on CPs.

The utilization of CPs as conductive filler/matrix is highly restricted due to the challenges in achieving effective dispersion, except PEDOT:PSS and polyaniline doped with bulky anions. Among them, only PEDOT:PSS possesses adequate conductivity to be employed as interconnects.^[^ ^169^ ^]^ PEDOT:PSS has also been blended with PDMS, Ecoflex, and PU to achieve various levels of mechanical stretchability. For example, composites of PEDOT:PSS containing SWCNTs, Ag NWs embedded in Ecoflex, and PU showed resistance‐insensitive stretchability and conductivity.^[^ ^170^ ^]^ Ag NWs based CP interconnects prepared by brush‐painting (Figure 6e) showed a stable resistance for 30 000 bending cycles at 1 mm bending radius and 10 000 twisting cycles at a twisting angle of 15°.^[^ ^164^ ^]^

Despite the fact that a few of the aforementioned examples did not directly employ the synthesized conductive materials for interconnect uses, their high conductivity (>1000 S cm^−1^) and strain‐insensitive characteristics grant them the potential to serve as viable interconnect materials. CP composites show flexibility and conductivity, however, their electrical performances are not comparable to metals‐based interconnects. Moreover, their performances are further compromised especially in cases that require stretchability too. To establish higher conductivity and stretchability, CP composites need secondary doping with elevated concentrations of toxic dopants, such as dimethyl sulfoxide and ionic liquids. The potential leakage of these hazardous materials on the human body greatly hinders their application in wearable bioelectronics. Therefore, further research and development are necessary to create stretchable and highly conductive CPs comprising non‐toxic components for biofriendly bioelectronics.

### 2.4 Carbon Materials

Carbon‐based materials have emerged as highly promising conductors in the field of wearable bioelectronics due to their exceptional mechanical, thermal, and biocompatible properties. Representative examples include carbon black (0D), CNT and carbon fibers (1D), graphene (2D), and their composites (3D). The structure and aspect ratio of carbon fillers, similar to metals, play a crucial role in determining the strain response of these conductive composites. While carbon black‐filled elastomers have been known for their conductivity for many years, their practical use as interconnects is limited. Their strong strain‐dependent electrical behavior makes them a more suitable candidate for strain‐sensing applications.^[^ ^171^, ^172^ ^]^ In contrast, CNTs and graphene have garnered substantial attention as interconnects owing to their outstanding attributes such as high conductivity, minimal toxicity, lightweight nature, and cost‐effective fabrication. Nevertheless, both CNT and graphene possess a honeycomb‐like crystal structure formed by sp^2^‐bonded carbon atoms, resulting in relatively low failure strains under stretching. CNTs are usually limited to 10% strain and graphene to 1% strain.^[^ ^173^, ^174^ ^]^ Compared to metal nanomaterials, carbon‐based nanomaterials exhibit relatively lower electrical conductivity but higher flexibility and transparency. They have demonstrated advantages in the formation of flexible structures by suppressing crack formation. Instead of pure CNT and graphene, in wearable electronics, they are exploited in the form of composite materials, combined with metals and polymers to achieve high conductivity and softness. Particularly, the high surface‐to‐volume ratios of CNTs and graphene allow significant enhancement in the conductivity of composites, even at low concentrations.^[^ ^175^, ^176^ ^]^

#### 2.4.1 CNT Composites

**Figure 7:**
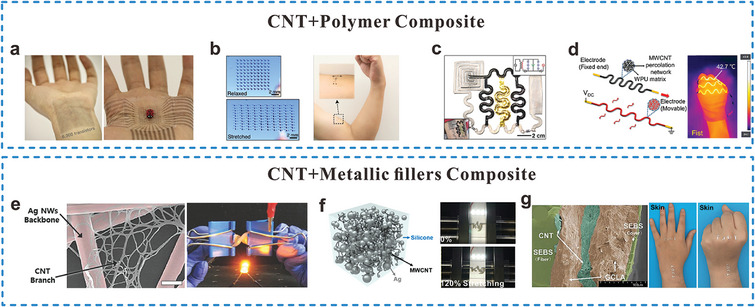
CNT‐based composites based interconnects for stretchable bioelectronics. a,b) stretchable skin electronics with CNT‐based transistor array and interconnects (Reproduced with permission.^[^ ^180^ ^]^ Copyright 2018, Macmillan Publishers Limited, part of Springer Nature. Reproduced with permission.^[^ ^181^ ^]^ Copyright 2021, Springer Nature), c) Wearable energy harvesting and storage devices with CNT‐PDMS conductors (Reproduced with permission.^[^ ^59^ ^]^ Copyright 2017, WILEY‐VCH), d) Serpentine stretchable conductors based on MWCNT/WPU for stretchable interconnects and wearable heaters (Reproduced with permission.^[^ ^60^ ^]^ Copyright 2023, Elsevier B.V.), e) Stretchable conductors formed with Ag NWs/CNT percolation networks, the scale bar is 300 nm (Reproduced with permission.^[^ ^182^ ^]^ Copyright 2014, WILEY‐VCH), f) Stretchable conductive adhesives for interconnects (Reproduced with permission.^[^ ^183^ ^]^ Copyright 2019, American Chemical Society), and g) Stable stretchable conductor with CNT and LM alternating layered structure (Reproduced with permission.^[^ ^184^ ^]^  Copyright 2024, Elsevier B.V.) are presented.

CNTs are categorized into single‐walled and multi‐walled CNTs depending on the number of graphene layer wrapped into a cylindrical form. SWCNTs have tuneable metallic properties through chirality variation, and MWCNTs own exceptional conductivity of up to 10^5^ S cm^−1^, making them ideal filler candidates.^[^ ^177^, ^178^, ^179^ ^]^ As shown in **Figure** **7**a–d, CNTs have been embedded in thermosetting and thermoplastic polymers such as PDMS, SEBS, and WPU for making conductive and stretchable interconnects. Specifically, CNT‐SEBS and CNT‐WPU composites were shown to have a stable electrical and mechanical performance under 100% strain.^[^ ^60^, ^180^, ^181^ ^]^ Serpentine‐patterned CNT‐PDMS interconnects showed negligible resistance change even after 1000 cycles of 50% strain.^[^ ^60^ ^]^

Conductivity of CNTs mainly relies on the atomic arrangement, alignment, and doping conditions. This introduces complexity in fabrication processes.^[^ ^59^, ^185^, ^186^ ^]^ An alternative approach to improve conductivity is the combination of CNTs with metal fillers to form a collaborative interconnected network. For example, a percolated network comprising Ag NW backbone and small CNTs achieved high electrical conductance, mechanical compliance, and transparency, endowing diverse applications, as shown in Figure 7e.^[^ ^182^ ^]^ The incorporation of a MWCNT/Ag particle percolated network within silicon adhesives yielded the electrical interconnects characterized by high electrical conductivity, durability, printability, and adhesiveness (Figure 7f).^[^ ^183^ ^]^ This conductive adhesive exhibited a high conductivity of 6450 S cm^–1^, and its conductivity was affected slightly after over 3000 cycles stretching at 50% strain. Recently, Gu et al. introduced CNTs into Ga–In–Cu LM to enhance the stability and adhesion of LM on elastomer substrates (Figure 7g).^[^ ^184^ ^]^ The CNT/LM‐modified SEBS fiber exhibited remarkable electrical conductivity of 1.43 × 10^4^ S cm^–1^ under extreme deformation of 1580% strain, with a resistance change of less than 3 Ω. Historically, CNT‐based interconnects show electrical performance decay as the structural changes in SWCNT networks caused by strain are frequently irreversible, resulting in inferior ambient stability compared to metal wires.^[^ ^187^ ^]^ Son et al. addressed this challenge by employing a self‐healing elastomer matrix, PDMS‐MPU_0.4_‐IU_0.6_, to encapsulate the conductive network composed of CNTs/Ag NWs.^[^ ^188^ ^]^ The self‐recovery of the elastomer enabled the reformation of conductive networks upon the formation of voids and exposures to wet environments, demonstrating promising prospects for applications in on‐skin long‐term monitoring.

#### 2.4.2 Graphene Composites

**Figure 8:**
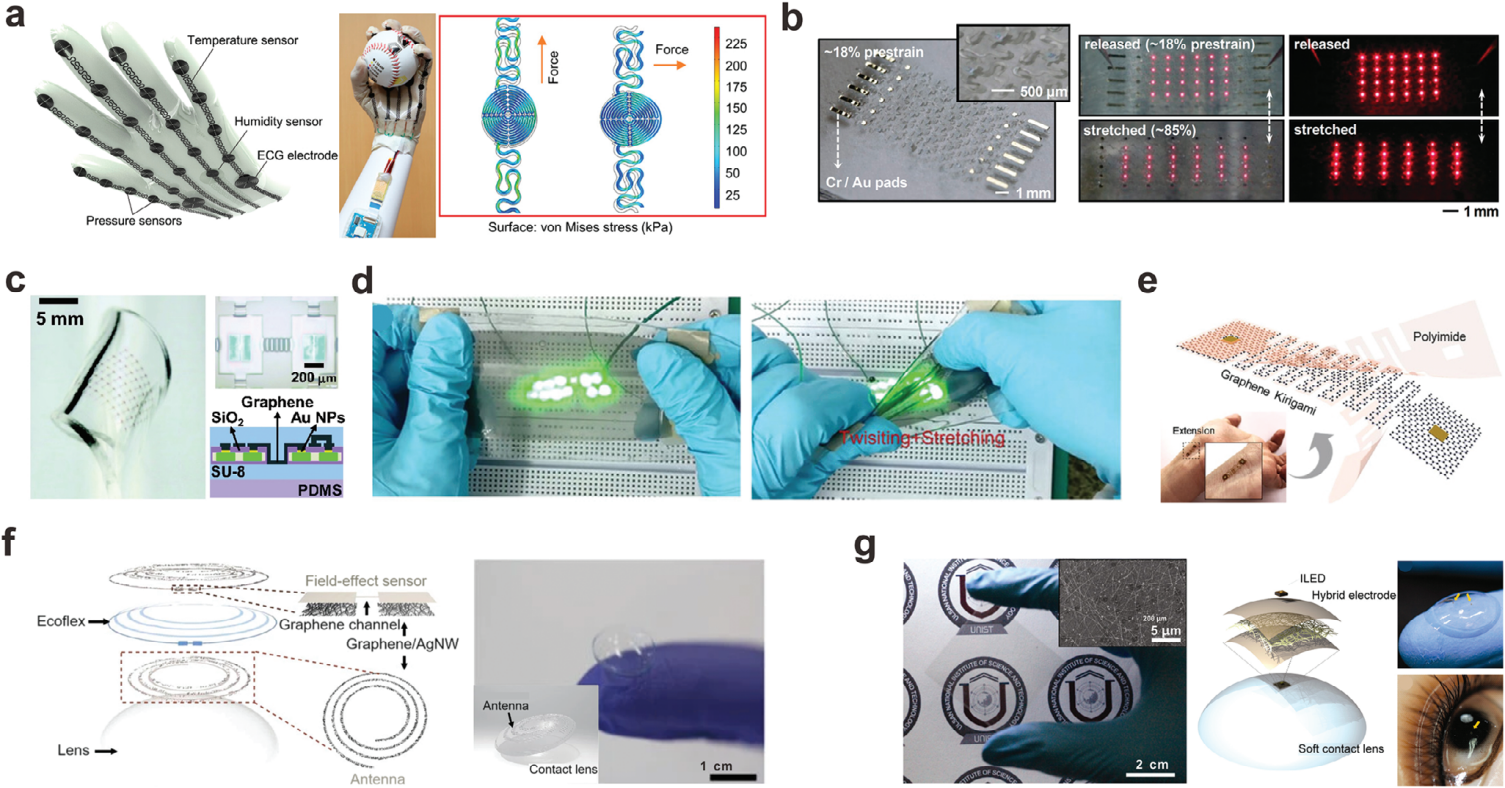
Stretchable electronics using graphene‐based conductors. a) Strain‐insensitive electronic glove with curved Graphene/CNT composite interconnects (Reproduced with permission.^[^ ^189^ ^]^ Copyright 2023, American Chemical Society), b) Stretchable and transparent graphene interconnects on PDMS substrate. (Reproduced with permission.^[^ ^190^ ^]^ Copyright 2011, American Chemical Society), c) stretchable Si‐based logic device with graphene/Au NPs interconnects. (Reproduced with permission.^[^ ^70^ ^]^ Copyright 2015, Wiley‐VCH) d) Strain‐insensitive sensor arrays based on wrinkled graphene‐elastomer composite (Reproduced with permission.^[^ ^57^ ^]^ Copyright 2021, Wiley‐VCH), e) Kirigami‐inspired sensor based on graphene (Reproduced with permission.^[^ ^69^ ^]^ Copyright 2019, Elsevier), f) Wearable smart contact lenses based on graphene/Ag NWs composite (Reproduced with permission.^[^ ^192^ ^]^ Copyright 2017, Springer Nature with a Creative Commons CC‐BY license), and g) Transparent and stretchable electrodes using graphene–metal NWs for displays on contact lenses (Reproduced with permission.^[^ ^193^ ^]^ Copyright 2013, American Chemical Society) are shown.

The mechanical mismatch between brittle graphene and stretchable substrate often leads to the formation of cracks, resulting in significantly increased resistance and potential electrical disconnections. To enhance the deformation tolerance in stretchable wearables, graphene, and graphene composites have been engineered into kirigami, mesh, and buckling structures (**Figure** **8**a–d). For example, a composite material comprising laser‐engraved graphene and CNTs with a ripple‐like meandering structure was utilized to achieve a strain‐insensitive interconnection for an e‐glove sensor, enabling the detection of pressure, temperature, humidity, and ECG signals.^[^ ^189^ ^]^ Similarly, stretchable and transparent graphene interconnects were deposited on PDMS with optimized noncoplanar serpentine shapes, showing stable electrical properties under 100% strain.^[^ ^190^ ^]^ Multilayer stacked graphene/Au NPs film with wrinkle structure served as interconnects for stretchable Si‐based logic devices.^[^ ^70^ ^]^ The thin film transistors connected by graphene interconnects showed high performance after being stretched by 10%. Also, wrinkled graphene/PDMS composite was used for developing strain‐insensitive interconnects for light‐emitting diode arrays, exhibiting negligible interference even under large strains.^[^ ^57^ ^]^ Furthermore, kirigami‐structured graphene interconnects encapsulated in Ecoflex and PDMS matrices demonstrated strain insensitivity under strains exceeding 200% (Figure 8e).^[^ ^69^, ^191^ ^]^ This conformal and breathable structure indicates the suitability of this platform for a wider range of on‐skin bioelectronics.

Incorporating metal NW fillers in graphene composites can offer not only high conductivity and stretchability, but also exceptional transparency, making it particularly suitable to be integrated into contact lenses as biomarker sensors and displays (Figure 8f,g). For instance, a graphene‐Ag NW hybrid was utilized to develop a multifunctional contact lens sensor for monitoring glucose levels in tears and intraocular pressure.^[^ ^192^ ^]^  Due to the large elasticity of graphene and mesh structure of Ag NW, the composite resistance remained almost constant (ΔR < 6%) after 5000 cycles of stretching at 25% tensile strain. By tuning the molecular binding on graphene, the sensor exhibited the capability to detect various disease‐associated biomarkers present in tear fluid. The same composite material has also been employed for the development of semiconductor and single‐pixel displays integrated into wearable soft contact lenses, highlighting the potential of using composite materials with 2D and 1D components for transparent and unobtrusive wearable sensors.^[^ ^193^ ^]^ The group demonstrated the potential of the hybrid material use as a  transparent and stretchable interconnects for soft bioelectronics.

Despite the extensive exploration of flexible electronics based on CNT and graphene, challenges related to uniformity, yield, and high cost persist, hindering the commercialization of carbon‐based wearable electronics.^[^ ^194^, ^195^ ^]^ Obtaining a large quantity of materials with the necessary purity remains a primary obstacle to implementing CNTs, as current production methods yield mixtures of semiconducting and metallic CNTs. Carbon materials also encounter challenges related to relatively high sheet resistance due to the randomly oriented network, multiple boundaries, and inherent defects.^[^ ^182^ ^]^ Moreover, the large‐scale synthesis of graphene ribbons poses significant challenges, particularly in terms of size control, further exacerbating the already complex fabrication process.^[^ ^176^ ^]^ Despite the mentioned limitations, leveraging the small dimensions of CNTs or graphene and integrating them strategically with biocompatible functional materials presents a promising strategy for enhancing the electrical performance of diverse devices.

## 3 Patterning Technologies

The implementation of complex structures and specialized materials necessitates the development of corresponding fabrication processes. Compared to traditional rigid device fabrication techniques such as photolithography and vacuum deposition, low‐temperature, large‐area, high‐throughput printing technologies are more suitable for flexible electronics manufacturing. In this regard, printing technologies, such as solution‐based printing, laser printing, and 3D printing, have been extensively researched and adopted for the mass production of flexible devices.

### 3.1 Solution‐Based Printing

Solution‐based printing has emerged as a promising approach for the large‐scale production of flexible electronics due to its cost‐effectiveness and high throughput. Various printing techniques, such as extrusion printer printing,^[^ ^196^ ^]^ inkjet printing,^[^ ^197^, ^198^ ^]^ screen printing,^[^ ^199^, ^200^ ^]^ and gravure printing,^[^ ^201^ ^]^ have been utilized to pattern multifunctional circuits. By optimizing ink solvents and matrices, it is possible to achieve circuits with high conductivity and adhesion without the need for high‐temperature processes. This enables the deposition of desired materials onto ultra‐thin polymer substrates, addressing issues such as signal errors and discomfort associated with rigid devices. Simple printing approaches, such as soft stamper‐based printing and bush painting techniques, provide a means to overcome the complexities of conventional fabrication methods while achieving high‐performance wearable electronics.^[^ ^164^, ^202^ ^]^

**Figure 9:**
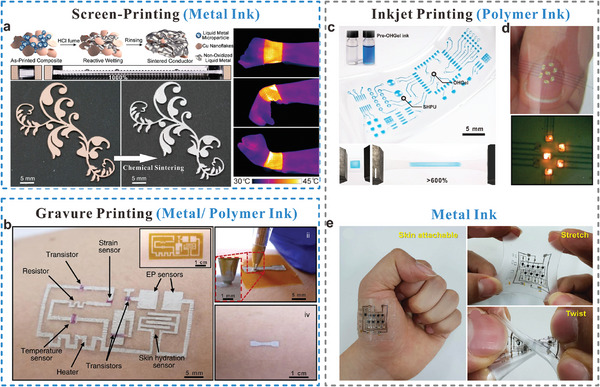
Solution‐based printing with tunable inks for stretchable bioelectronic applications. a) Screen‐printed LM/microparticle ink for ultra‐stretchable wearable electronics (Reproduced with permission.^[^ ^204^ ^]^ Copyright 2020, American Chemical Society), b) Drawn‐on‐skin electronics with metal and polymer inks (Reproduced with permission.^[^ ^205^ ^]^ Copyright 2020, Springer Nature with a Creative Commons CC‐BY license), c) Inkject‐printed supramolecular gel‐elastomer system (Reproduced with permission.^[^ ^206^ ^]^ Copyright 2023, Springer Nature with a Creative Commons CC‐BY license), d) Printing of CP for intrinsically stretchable interconnects and circuits (Reproduced with permission.^[^ ^207^ ^]^ Copyright 2019, WILEY‐VCH), and e) Stretchable strain‐tolerant soft PCB (Reproduced with permission.^[^ ^208^ ^]^ Copyright 2019, Taylor & Francis) are demonstrated.

To minimize resistive energy loss, high‐thickness deposition techniques like screen printing and stencil printing are often employed for the interconnects.^[^ ^183^, ^203^ ^]^ For instance, Li et al. utilized a screen‐printing technique to pattern 70 µm thick LM composite interconnects onto an elastomer substrate, followed by a chemical sintering process to eliminate passivating oxide layers, as shown in **Figure** **9**a.^[^ ^204^ ^]^This approach enabled the achievement of high conductivity (>10^4^ S cm^−1^) and stretchability exceeding 1000%. Ershad et al. demonstrated the fabrication of ultra‐conformal, customizable, and deformable electronics through direct stencil printing of Ag‐PEDOT:PSS ink onto the skin (Figure 9b).^[^ ^205^ ^]^ By repeatedly applying the printing process to the same location, they successfully achieve conductive layers with varying thicknesses ranging from a few hundred nanometers to 10 µm. The printing process allowed for the on‐demand creation of devices in a freeform manner, showcasing the versatility of the approach for the fabrication of complex and multifunctional electronic systems.

On the other hand, inkjet printing serves as a maskless deposition technique that conserves materials and enables precise development of conductive lines with notable complexity.^[^ ^197^ ^]^ In comparison to screen printing or gravure printing methods, which typically have a minimum dot size of 20 µm, inkjet printing provides the capability to achieve well‐defined patterning (e.g., Au NPs interconnects) with submicron resolution, while preserving high conductivity (∼210^4^ S cm^−1^).^[^ ^209^ ^]^ The solution‐based printing process also offers the ability to print CP inks, enabling the fabrication of inherently stretchable fully organic electronics. As shown in Figure 9c, Gao et al. inkjet‐printed ionic organohydrogel with sophisticated geometries, which was robustly interfaced onto hierarchically H‐bonded PU.^[^ ^206^ ^]^ Kraft et al. fabricated highly conductive PEDOT:PSS interconnects via inkjet printing as well, as shown in Figure 9d, enduring strains above 100% and exhibiting excellent stability in air (less than 5% resistance change over 1 month).^[^ ^207^ ^]^ Inkjet printing techniques have demonstrated their capability to fabricate a strain‐tolerant soft circuit board by producing both wrinkled structure metal interconnects and rigid epoxy patterns.^[^ ^208^ ^]^ The prototype circuits demonstrated consistent operational performance when subjected to diverse mechanical deformations, including the 180° folding state and irregular deformed state with a tensile strain of 25% (Figure 9e). These highlight the resilience and adaptability of the inkjet‐printed circuits, suggesting that it is a viable method for creating flexible and mechanically robust wearable electronic systems.

### 3.2 3D Printing

Diverging from conventional semiconductor fabrication processes, stretchable devices typically exhibit a low‐density single‐layer configuration due to the limitations imposed by flexible substrates and patterning techniques. 3D printing offers a promising approach to enhance the complexity and design freedom of electronic devices by enabling the formation of arbitrary 3D structures.^[^ ^210^ ^]^ In a fully 3D printed system, different functional layers such as interconnects, isolation layers, and electrodes can be fabricated using various printing materials and deposition methods.^[^ ^211^, ^212^ ^]^ For interconnects in particular, self‐standing conductive pastes are commonly used to prevent unwanted flow during the printing process caused by gravity or substrate slope.^[^ ^213^ ^]^ By adjusting the ink viscosity and the adhesion strength at the ink‐substrate interface, 3D printing technology shows the feasibility of producing complex, highly integrated stretchable devices under low‐temperature conditions.

**Figure 10:**
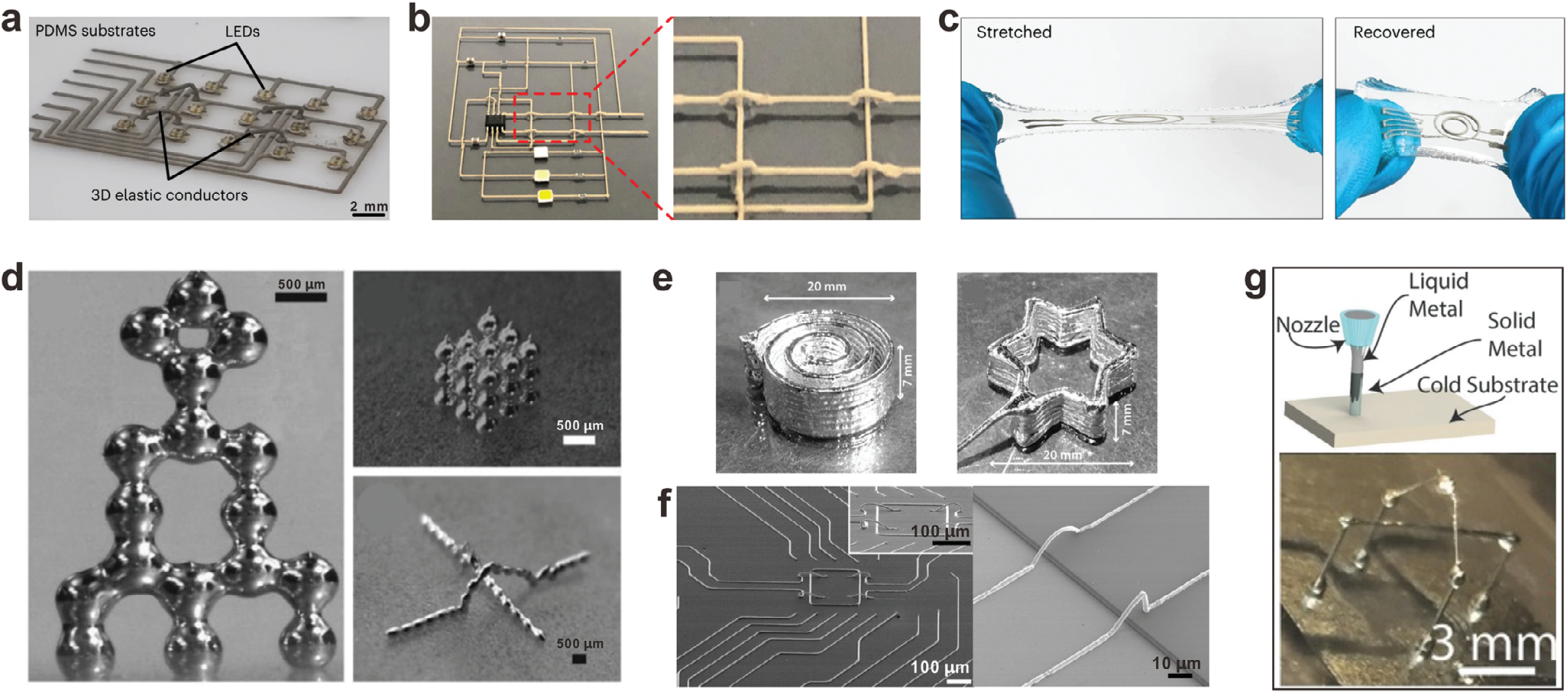
3D printed interconnects. a) Omnidirectional printed 3D elastic conductors using polymer/Ag particle/MWCNT composite ink (Reproduced with permission.^[^ ^214^ ^]^ Copyright 2023, Springer Nature), b) 3D circuit printed using elastic silver ink (Reproduced with permission.^[^ ^213^ ^]^ Copyright 2022, Wiley‐VCH), c) Printing 3D interconnects in hydrogel (Reproduced with permission.^[^ ^215^ ^]^ Copyright 2022, Springer Nature), d) 3D printing of free‐standing LM microstructures (Reproduced with permission.^[^ ^216^ ^]^ Copyright 2013, WILEY‐VCH), e) LM conductive lines with modified rheology (Reproduced with permission.^[^ ^217^ ^]^ Copyright 2018, WILEY‐VCH), f) High‐resolution LM with 3D structures (Reproduced with permission.^[^ ^218^ ^]^ Copyright 2019, American Association for the Advancement of Science), g) Freeze‐printing of LM alloys for 3D conductive networks (Reproduced with permission.^[^ ^219^ ^]^ Copyright 2016, WILEY‐VCH) are exhibited.

To address structural collapse and nozzle clogging during the extrusion process, Lee et al. developed a printable ink formulation that incorporates a conductive elastomer composite, immiscible solvent, and emulsifying solvent.^[^ ^214^ ^]^ As shown in **Figure** **10**a, the resulting elastic conductors exhibited freestanding, filamentary, and out‐of‐plane 3D structures with diverse geometries, achieving a feature size smaller than 100 um. Furthermore, these conductors demonstrated a high conductivity of up to 6682 S cm^−1^ and a remarkable stretchability exceeding 150%. Similarly, Sun et al. adjusted the rheological characteristics of silver paste to enable the creation of vertical interconnect accesses on elastomeric substrates (Figure 10b).^[^ ^213^ ^]^ A protective core‐shell structure was formed by printing rigid PDMS around the specific region of the interconnect, enabling a stable electrical response of the entire device during hundreds of bending and stretching/releasing cycles at the tensile strain of 0–40%.

Furthermore, the 3D‐printed interconnects can also be reinforced by the surrounding matrix. An example is the utilization of granular ionically crosslinked hydrogel, which can exhibit a fluidic behavior in response to shear stress caused by nozzle movement.^[^ ^215^ ^]^ After the deposition of conductive ink, the hydrogel can be transitioned to a solid state. As shown in Figure 10c, the ink is encapsulated within the monolithic hydrogel, resulting in a soft and stretchable interconnect with a high conductivity of 1.4 × 10^3 ^S cm^−1^.

Interestingly, LM‐based inks are compatible with 3D printing technology as LM forms a thin oxide layer in the presence of air, providing sufficient solidity to retain its shape against the forces of gravity and surface tension. This allows for the self‐supported LM trace by stacking the droplets or extruding ultrathin filaments (Figure 10d,e).^[^ ^216^, ^217^ ^]^ Park et al. achieved high‐resolution LM 3D structures with a minimum line width of only 1.9 µm by combining precise nozzle control and support from oxide skin (Figure 10f).^[^ ^218^ ^]^ This high‐resolution 3D fabrication approach can be combined with conventional manufacturing techniques for developing highly integrated stretchable wearable devices. It is worth noting that unlike conductive inks that solidify through solvent evaporation, LM can undergo a transition to a solid state at low temperatures. This unique property enables the freeze‐printing of LM alloys, facilitating the creation of self‐supporting horizontal lines between vertical supports, as shown in Figure 10g.^[^ ^219^, ^220^ ^]^

### 3.3 Laser Patterning

Laser patterning processes offer remarkable advantages over conventional photolithography methods in terms of precise patterning. These processes offer high printing speed and resolution. Most importantly, they can be performed under ambient conditions without the need for a controlled atmosphere or clean room environment, thereby enabling large‐scale and rapid production.^[^ ^221^ ^]^ Laser patterning techniques including laser cutting, laser scribing, laser‐induced forward transfer, and laser sintering have been established as crucial fabrication methods for flexible electronic products. In particular, the utilization of short and ultrashort laser pulses in laser processing enables maskless and selective irradiation leading to minimization of heat‐affected zones, thereby achieving lateral resolutions below 1 µm.^[^ ^222^ ^]^ Moreover, the intensity of the laser pulse can be adjusted to meet the specific requirements of each pattern.^[^ ^223^ ^]^

**Figure 11:**
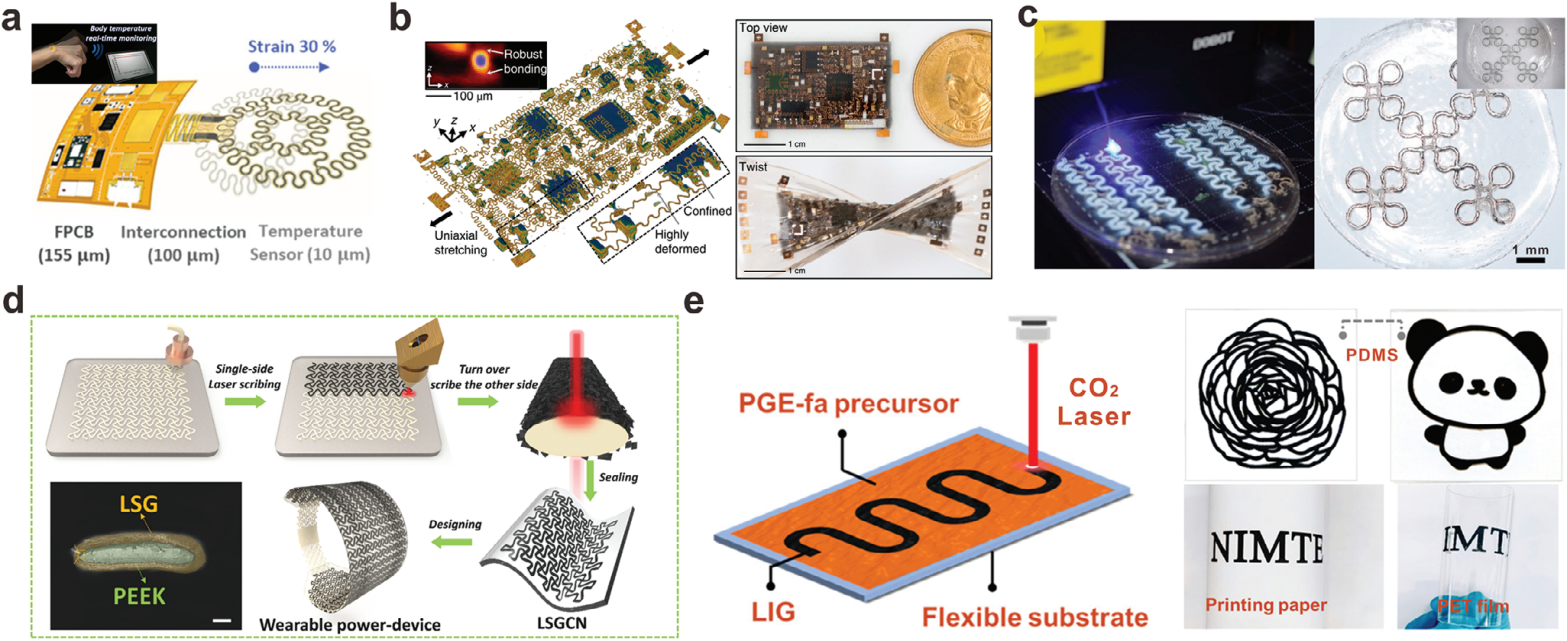
Stretchable interconnects based on laser printing. a) Laser‐processed stretchable‐gradient interconnection for temperature sensor (Reproduced with permission.^[^ ^225^ ^]^ Copyright 2021, American Chemical Society), b) 3D integrated stretchable electronics based on laser‐printed circuits (Reproduced with permission.^[^ ^226^ ^]^ Copyright 2018, Springer Nature with a Creative Commons CC‐BY license), c) Laser‐engraved LM circuit for wearable electronics (Reproduced with permission.^[^ ^227^ ^]^ Copyright 2022, MDPI with a Creative Commons CC‐BY license), d) Stretchable core‐shell laser scribed graphene conductive network (Reproduced with permission.^[^ ^228^ ^]^ Copyright 2022, Elsevier), e) In‐situ growth of laser‐induced graphene for wearable electronics (Reproduced with permission.^[^ ^229^ ^]^ Copyright 2024, American Chemical Society) are demonstrated.

Laser cutting technology has been widely applied to pattern conductors on flexible substrates, allowing the development of diverse designs to enhance the performance of wearable devices. For example, the Au film deposited on PI could be laser patterned into open‐mesh architectures with a coverage ratio of 30%. These structures ensured conformal contact with dynamic skin and facilitated the permeation of sweat, thereby promoting comfort and functionality in wearable electronics.^[^ ^224^ ^]^ Kang et al. utilized laser cutting to integrate kirigami and serpentine structures with varying substrate thickness, resulting in the development of a stretchable‐gradient interconnection.^[^ ^225^ ^]^ As shown in **Figure** **11**a, this approach enables the acquisition of highly accurate and long‐term human health information during dynamic environmental situations. Huang et al. employed a laser system to create copper interconnect networks and vertical interconnect accesses, leading to the development of a 3D stretchable electronics platform (Figure 11b).^[^ ^226^ ^]^ This framework offered a significant advancement in device integration capabilities and opened up possibilities for the development of devices with higher complexity in interconnect design. As shown in Figure 11c, laser techniques can also be employed to create grooves on elastomer or paper‐based substrates, which can be filled with conductive LMs to achieve micron‐scale conductive interconnects.^[^ ^227^ ^]^

In terms of printed conductive interconnects, the sintering process is one of the determining factors for grain morphology and uniformity, which can be controlled through laser sintering by adjusting the parameters.^[^ ^230^ ^]^ In contrast to conventional sintering methods like ovens, laser sintering utilizes photon‐induced sintering, rapidly and intensively inducing phase transitions in the irradiated regions without subjecting the devices to high temperatures. This feature makes laser sintering particularly suitable for wearable electronics based on flexible substrates.^[^ ^231^, ^232^ ^]^

Apart from metal interconnects, laser scribing of conductive materials has attracted significant research interest in recent years.^[^ ^233^ ^]^ By locally inducing polymer photothermal pyrolysis using a CO_2_ laser source, patterned porous graphitic carbon structures can be formed, known as laser‐induced graphene. The entire process can be performed in ambient air, directly written on carbon materials, and does not require any solvents, making it highly attractive for industrial applications.^[^ ^234^ ^]^ Due to its porous structure, laser‐induced graphene can easily combine with various elastic polymer precursors to form composites for the fabrication of flexible conductors (Figure 11d).^[^ ^228^, ^235^ ^]^ Alternatively, organic coatings can be applied to elastic substrates, and conductive pathways can be induced in situ on the elastic base using laser irradiation, as illustrated in Figure 11e.^[^ ^229^ ^]^

The advancement of flexible and wearable electronics necessitates the exploration of innovative fabrication techniques. Researchers have made significant progress in developing various patterning methods to fulfill the requirements of ink and substrate materials. Examples include the use of magnets to manipulate the motion of conductive inks and the introduction of chemical/laser processes for selective adhesion of the inks.^[^ ^236^, ^237^ ^]^ However, the stability and density of circuits on soft substrates are still limited due to mechanical mismatches and the space needed for stretchable structure designs. In addition to the printing methods covered, other patterning approaches such as molding transfer, transfer printing, and chemical vapor deposition (CVD) are also widely employed in the manufacturing of wearable devices. These patterning methodologies are predicated on preceding patterning techniques, such as the fabrication of molds, stamp‐based patterning, and the chemical vapor deposition of graphene on pre‐patterned metal surfaces. As the underlying principles governing these patterning methodologies do not directly determine the patterning of the conductive materials, they do not constitute the primary focus of discussion within this section.

## 4 I/O Interface

The I/O interface plays a critical role in wearable electronics as it can directly affect the overall signal integrity. In wearable devices, materials with low modulus similar to the epidermis are commonly used for conformal contact with the skin and for the acquisition of weak physiological signals at a high resolution as presented in earlier sections. These materials are lightweight, thin, and stretchable to enhance the imperceptibility of the sensors. However, in integrated systems, the thin and soft components are often accompanied by more rigid signal conditioning, processing, and transmission systems to translate the physiological signals into readable biological information. Therefore, achieving a reliable and stable connection between soft and rigid materials is necessary. Although numerous efforts have been made to introduce different types of interconnect materials and structures to capture these weak signals,^[^ ^238^, ^239^ ^]^ mechanical mismatch at the I/O interface is often overlooked. A significant mechanical mismatch between the interconnect and rigid components results in stress concentration at the interface, leading to large noises and subsequent failure of the devices.^[^ ^240^ ^]^ Therefore, the development of high‐quality connections between stretchable and rigid electronics is crucial.

Traditional electrical connections such as zero insertion force (ZIF) connectors, snap fasteners, and soldering are widely utilized approaches in the electronic industry for their stable and efficient signal transfer to PCBs. However, these connectors have notable limitations in compatibility with flexible devices. ZIF connectors, designed for easy insertion and removal of ICs, require the insertion pins to possess a certain strength for a firm grip in the ZIF sockets.^[^ ^241^ ^]^ However, the clamping force exerted by the sockets causes significant deformation in the flexible material, resulting in unstable connections. Moreover, during dynamic body movements, the rigid sockets can lead to large friction and permanent wear, shortening the longevity of the flexible devices. Additionally, high‐temperature soldering can easily damage the polymer substrate and is incompatible with carbon conductors. Snap fasteners require non‐stretchable and robust areas on the device for electronic connection. To attain a more compatible I/O interface, simple approaches using conductive pastes and anisotropic conductive films have been explored.^[^ ^234^ ^]^ However, these solutions are constrained by their lack of stretchability and their dependence on high‐temperature or pressure processing, which may not be suitable for flexible device fabrication. Currently, researchers employ two primary strategies to establish robust connections for flexible and stretchable devices. One approach involves the synthesis of soft connections,^[^ ^33^, ^242^ ^]^ and the other approach focuses on introducing energy dissipation layers between the soft interconnects and rigid electrical components.^[^ ^208^ ^]^ In this section, we will introduce these two strategies and some recent advances in the design of robust interfaces for stretchable electronics.

### 4.1 Soft Connection Strategy

**Figure 12:**
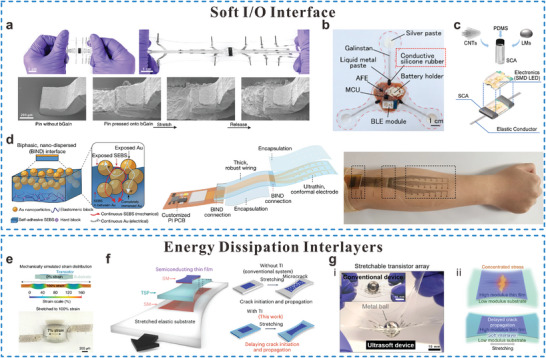
Strategies for a stable interfacial soft‐rigid connection in stretchable bioelectronics. a) Soft interfacial connection using biphasic LM (Reproduced with permission.^[^ ^32^ ^]^ Copyright 2021, Springer Nature), b) Liquid‐solid heterconnector for robust connection on transformable ECG patch (Reproduced with permission.^[^ ^33^ ^]^ Copyright 2021, American Chemical Society), c) Stretchable conductive composite adhesives for soft connection in wearable electronics (Reproduced with permission.^[^ ^242^ ^]^ Copyright 2020, Elsevier), d) A universal connection interface for stretchable devices (Reproduced with permission.^[^ ^244^ ^]^ Copyright 2023, Springer Nature), and e–g) Energy dissipation interlayers for stretchable electronics (Reproduced with permission.^[^ ^181^ ^]^ Copyright 2021, Springer Nature. Reproduced with permission.^[^ ^246^ ^]^ Copyright 2022 Springer Nature. Reproduced with permission.^[^ ^247^ ^]^ Copyright 2023, Springer Nature with a Creative Commons CC‐BY license) have been developed.

The essence of this soft connection strategy is the integration of a malleable active material with rigid components. This strategy enables the active layer to adapt to the deformation while maintaining connection with rigid structures, precluding the introduction of failure‐inducing stress concentrations and delamination. For example, solid‐liquid biphasic GaIn alloy was employed not only as interconnects but also as vertical contact points to maintain robust connections with rigid electrical components under highly demanding conditions (**Figure** **12**a).^[^ ^32^ ^]^ In this biphasic layout, the rigid pin breaks the solid oxide layer during deformation, allowing LMs to flow in and encapsulate the soft‐rigid contact area. The solid particles with strong wettability help LMs to anchor and adhere to the pin, thereby ensuring stable I/O connections. Solid–liquid composites consisting of adhesive polymers and LM can also provide high interfacial adhesion energy and efficient energy dissipation at the I/O interface, allowing robust bonding with IC chips under biaxial stretching. Similarly, hybrid LM (a combination of partially oxidized LM and LM) can work as a solder to connect electronic chips on a highly stretchable fibrous SBS substrate. The partially oxidized LM was chosen to provide good adhesion between the LM circuits and the rigid pins. This solder showed negligible resistance change when the circuit was stretched to 1500% strain.^[^ ^243^ ^]^ Takaya et al. introduced a novel approach involving a heteroconnector that combines solid‐liquid paste and conductive silicone rubber.^[^ ^33^ ^]^ As shown in Figure 12b, this heteroconnector enabled the gradual connection of LM interconnects with a rigid PCB without compromising the overall stretchability of the system. Similarly, Dou et al. developed a stretchable conductive adhesive by incorporating PDMS, LMs, and CNTs (Figure 12c).^[^ ^242^ ^]^ The resulting adhesives exhibited excellent stretchability, electrical conductivity, stability, and strong adhesion to polymer substrates. Moreover, they exhibited stress‐buffering capabilities by redistributing the localized stress at the electronics interface to a larger proximity, which effectively prevented circuit breakage during deformation and significantly enhanced the overall performance of the devices. In addition, the use of self‐adhesive or self‐healing polymer matrices with interpenetrating metal NPs or NWs on the surface can provide high interfacial toughness and serve as electrical pathways.^[^ ^244^, ^245^ ^]^ As shown in Figure 12d, this approach has been reported to create a universal interface, where interpenetrating Au NPs are embedded within a SEBS matrix to form a robust and stretchable connection in a plug‐and‐play manner.^[^ ^244^ ^]^ These properties render the soft connectors highly suitable for applications requiring stretchable multilayer connections such as wearable devices and soft robots.

### 4.2 Energy Dissipation Strategy

Alternatively, an energy dissipation layer can be positioned between the rigid counterparts and flexible substrates to mitigate the strain concentration at the rigid/soft interface.^[^ ^248^ ^]^ This approach aims to prevent the interface from an abrupt strain change and hence, minimize potential damage. In general, tough polymer materials can be employed as the energy dissipation layer as they can be easily bonded with the substrate. For example, Wang et al. introduced patterned regions of mechanical heterogeneity into elastomer substrates by selectively modulating the cross‐linker's density. This generated localized regions of high and low stiffness and reduced strains on the active regions of the device (Figure 12e).^[^ ^181^ ^]^ Similarly, Kang et al. proposed a universal design for mitigating delamination and strain‐induced crack initiation and propagation on semiconducting thin films (Figure 12f).^[^ ^246^ ^]^ This strategy involved the incorporation of a tough, self‐healing polymer matrix, which could repetitively dissipate energy through autonomous and dynamic bond breakage and reformation. The surface modifier which formed covalent and non‐covalent bonds with the tough polymer and solely covalent bonds with the elastic substrate strengthened the interface between the semiconducting thin film and the substrate, effectively enhancing the mechanical integrity of the semiconducting thin film. On the other hand, an intermediate medium with a compromised modulus has been proposed to minimize the difference between high‐modulus functional components and a low‐modulus stretchable substrate.^[^ ^247^ ^]^ As shown in Figure 12g, the implementation of such an intermediate layer facilitated the development of stretchable transistors and active matrices with sub‐10 kPa moduli. Consequently, the resulting structures exhibited enhanced conformability and reduced mechanical constraints when they were attached to irregular and deforming skin and tissue surfaces.

The energy dissipation design strategy can also be applied to the vertical interface connection of high‐density integrated stretchable electronics. To connect multilayer interconnects or chips, vertical interconnect access and interface connections are required.^[^ ^249^ ^]^ Traditional PCBs achieve this through drilled and metalized holes. However, for stretchable devices, drilling can easily induce deformation of the elastomeric materials, leading to misalignment or damage. Furthermore, due to the disparate constructions of each device layer, the degree of deformation under strain varies across different locations.^[^ ^226^ ^]^ In this context, rigid vertical interconnects are susceptible to shear‐induced failure.

By strategically incorporating localized energy dissipation layers, core‐shell structured vias can be fabricated to mitigate the strain experienced by the via structures and their internal interconnect materials. This energy dissipation design approach enables the realization of robust vertical interconnects that can accommodate the heterogeneous deformation profiles inherent to high‐density integrated stretchable electronic systems.^[^ ^213^, ^250^ ^]^

Despite the limitations posed by a restricted integration density compared to conventional rigid ICs or the requirement for pre‐treating the counterparts or substrates, these interfacial connections offer significant avenues for enabling the practical implementation of stretchable wearable electronics.

## 5 Conclusion

Stretchable interconnects are indispensable components of wearable bioelectronics. Herein, we summarized the recent progresses in the research and development of the interconnects, including the materials, geometrical arrangements, form factors, and manufacturing technologies to achieve superior electrical conductivities and mechanical robustness for reliable I/O interfaces and signal acquisitions.

Structured metal films are the most commonly utilized form of interconnects as they guarantee high electrical conductivity without significant decline in resistance under small structural deformation. Yet the brittleness hinders their stable electrical connection and mechanical robustness at large strains. The larger the strains, the larger the spatial arrangements are needed to accommodate buckling, thereby limiting the device density and introducing additional design complexities. To achieve more precise control over the formation of buckling structures, the substrate is required to possess a certain minimum thickness to attain the requisite modulus. Consequently, the thicknesses of the substrate and encapsulation layers often substantially exceed that of the conductive elements, thereby constraining the overall conformability and compliance of the device. Therefore, structured metals are more popular for certain applications wherein the electrical performance is a priority for efficient signal acquisition, and/or the devices only experience relatively small strains. LMs address these limitations by their innate stretchability and self‐healing ability. Despite possessing lower conductivity than solid metals, they can be easily modified to desired structures, and their strain‐responsive electrical property can be effectively tuned by tailoring the encasing architectures. This makes LMs ideal for devices undergoing large and dynamic strains. Nevertheless, their liquid nature and the requirement of encasing substrates demand delicate surface modifications to obtain strain‐tolerant, structurally adherent, and high‐performing interconnects. These strenuous fabrication methods limit the widespread adoption and large‐scale production of LMs. In addition, the potential leakage of LMs as a result of incompatible sintering and sealing methods further hinders their development. Therefore, simple and generalizable processing methods are essential to fully realize the potential LM‐based interconnects for stretchable wearable sensors.

Inherently stretchable CPs offer the advantages of intrinsic stretchability, conductivity, and biocompatibility. However, the majority of CP‐based interconnects are primarily limited to PEDOT. Although their conductivity is not as high as the metal materials, it can be enhanced by controlling the crystallinity of conjugated polymers. A major drawback of CPs is the trade‐off between the stretchability and conductivity. The addition of conductive fillers has been extensively explored to achieve improved conductivity and maintain a certain extent of structural disorder for stretchability. In the future, non‐toxic and highly conductive additives that promote the formation of continual conductive domains and enhanced stretchability, can be explored for developing CP‐based interconnects. Alternatively, composites based on carbon‐based fillers can be combined with elastomeric matrices to create stretchable conductive pathways. However, the trade‐off resulting from stiffness arising from high filler concentrations and the high resistance arising from low filler concentrations still needs to be addressed. Therefore, novel and innovative material development strategies that can parallelly offer high conductivity, stability, and stretchability are highly anticipated.

Among the three material categories, metallic conductors exhibit the highest electrical conductivity but suffer from relatively elevated costs. Carbon‐based materials possess excellent chemical stability and flexibility; however, the realization of their high electrical conductivity necessitates sophisticated control and specialized fabrication methods. On the other hand,C offer the advantages of high transparency and solution processability, yet their electrical conductivity and material selection range remain limited. Distinct from the individual characteristics of these singular material classes, polymer‐based composite materials exhibit exceptional compatibility with a wide range of solution‐based fabrication methods, in addition to possessing a remarkable degree of tunability in their properties. These attributes render polymer‐based composites as promising candidate materials with the potential for widespread adoption and utilization.

In addition to the materials and their form factors, patterning and fabrication techniques play a crucial role in the development of desirable interconnects for wearable devices. Considering the thermally delicate polymer substrates, fabrication with low pressure, room temperature, and non‐destructive process is very important for transitioning the devices from the laboratory to real‐world use.^[^ ^251^ ^]^ Rapid patterning techniques such as laser printing and 3D printing demonstrate remarkable advantages over conventional photolithography, providing opportunities for large‐scale productions of wearable electronics and implementation in industrial scale. Beyond scalability, these tunable techniques offer control over the adhesion between the conductive ink and substrates which may lead to further enhancement of interfacial stability. The mechanical stability and durability of the stretchable interconnect still need to be extensively exploited for long‐term wearable applications. For instance, the mechanical characteristics and deformations, i.e., flex cycles, frequencies, and bending radius of human skin differ by location and across individuals, so it is critical to carefully assess how the developed bioelectronics can meet those specific needs for intended applications. Concurrently, the utilization of toxic solvents during the fabrication process, as well as the subsequent hygiene issues, necessitate heightened attention and careful consideration in the development of bio‐integrated devices. The potential environmental and human health implications associated with these factors must be thoroughly addressed and mitigated in the pursuit of such applications.

In addition, compared to their rigid counterparts, dynamic wearable electronics are still limited in terms of accuracy, robustness, durability, and reliability. Current research has primarily focused on enhancing the performance of individual components or subsystems, rather than addressing the overall stability and reliability of the integrated device. For example, the current generation of wearable sensors often features only a soft sensing layer, which becomes rigid after integration with PCBs. Moreover, the development of shielding layers akin to traditional wiring, as well as various electronic components with comparable levels of softness, remains lacking. In particular, more efforts are needed to address the mismatches in I/O interface for the realization of a truly soft and fully integrated stretchable system.

As such, the translational applications of stretchable conductors for future wearable electronics should not solely focus on optimizing material properties to achieve unnecessarily heightened sensitivity and signal strength. Rather, the emphasis should be placed on the following system‐level considerations:

i) The chemical stability and biocompatibility of the composite materials are necessary to ensure their safety, reliability, and high‐performance operation in challenging environments such as on the wounds, during perspiration, and under thermal environments.
ii) The spatial arrangement of the stretchable interconnect geometries needs to be carefully devised to achieve high spatial resolutions exceeding the requirements of the biological tissues. As the deformable geometries require space, the stretchable electronics are typically in low‐density and single‐layer formats. The adoption of multi‐layer and 3D integration strategies represents promising approaches for high‐density electronics fabrication.
iii) A firmly attached interface with signal processing systems should be developed to guarantee the robust integration and long‐term stability of the devices.
iv) Scalable manufacturing processes are needed for high‐throughput and cost‐efficient production to translate the technologies from academic research into the industry.

These multifaceted criteria must be holistically addressed to enable the widespread adoption and realization of advanced wearable sensor technologies.

In summary, recent breakthroughs on stretchable and wearable bioelectronics have yielded great potential for applications in healthcare monitoring. However, overcoming the above‐mentioned technical challenges is key to meeting the increasing demand for wearable health technology and realizing accurate remote health assessment. As stretchable bioelectronics is advancing towards highly integrated and intelligent systems, it is foreseeable that soft bioelectronics will be combined with complex sensing feedback and intelligent classification systems, offering significant prospects for personalized healthcare.

## Conflict of Interest

The authors declare no conflict of interest.
